# Bayesian Network Meta-Analysis of Long-Term Survival: Single vs Multiple vs Total Arterial Coronary Bypass Grafting

**DOI:** 10.1016/j.jacadv.2026.103178

**Published:** 2026-08-09

**Authors:** Justin Ren, Christopher M. Reid, David H. Tian, Christopher Siderakis, Julian A. Smith, Colin Royse, Andrea Bowyer, Ximena Cid-Serra, Winnie Zhang, Gina Arena, Alistair Royse

**Affiliations:** a Department of Surgery, The University of Melbourne, Melbourne, Australia; b Department of Cardiothoracic Surgery, Royal Melbourne Hospital, Melbourne, Australia; c Medical School, The University of Western Australia, Perth, Australia; d Population Health, Curtin University, Perth, Australia; e School of Public Health and Preventive Medicine, Monash University, Melbourne, Australia; f Department of Anesthesia and Perioperative Medicine, Westmead Hospital, Sydney, Australia; g Department of Surgery, Monash University, Melbourne, Australia; h Department of Cardiothoracic Surgery, Monash Health, Melbourne, Australia; i Department of Anesthesia and Pain Management, The Royal Melbourne Hospital, Melbourne, Australia; j Outcomes Research, University of Texas Health, Houston, Texas, USA

**Keywords:** Bayesian network meta-analysis, coronary artery bypass grafting, multiple arterial grafting, survival analysis, total arterial revascularization

## Abstract

**Background:**

Arterial conduit strategies in coronary artery bypass grafting (CABG) have been associated with superior graft durability to saphenous vein grafts. However, the relative benefits of multiple arterial grafting (MAG) and total arterial revascularization (TAR) remain uncertain due to limited direct comparative evidence.

**Objectives:**

This systematic review and Bayesian network meta-analysis (PROSPERO; CRD420251117898) evaluated long-term survival outcomes associated with single arterial grafting (SAG), MAG, and TAR.

**Methods:**

Medical Literature Analysis and Retrieval System Online, Excerpta Medica Database, and the Cochrane Library were searched from inception to March 2026 for studies comparing CABG conduit strategies reporting adjusted HRs for long-term all-cause mortality. Studies including adults undergoing isolated CABG with SAG, MAG, or TAR were eligible. A Bayesian random-effects network meta-analysis was performed to integrate direct and indirect evidence. This framework permitted quantification of TAR-versus MAG comparisons, which had previously been limited by scarce direct evidence. Treatment ranking was assessed using the surface under the cumulative ranking curve.

**Results:**

Fifty-four studies including 560,723 patients were analyzed. Compared with SAG, MAG was associated with lower long-term mortality (HR: 0.83; 95% credible interval [CrI]: 0.79-0.85), whereas TAR demonstrated a greater survival benefit (HR: 0.75; 95% CrI: 0.70-0.79). Indirect comparison within the network suggested an incremental survival advantage of TAR over MAG (HR: 0.90; 95% CrI: 0.85-0.96). Treatment ranking suggested a hierarchy in which TAR was associated with superior survival, followed by MAG and SAG.

**Conclusions:**

Increasing use of arterial conduits during CABG is associated with progressively improved long-term survival, with TAR demonstrating the greatest benefit.

Coronary artery bypass grafting (CABG) is preferred over percutaneous coronary intervention for the treatment of multivessel or left main coronary artery disease.^1^, ^2^, ^3^ This preference may be attributed to CABG's ability to establish a new conduit for blood flow to the distal anastomotic site, rather than merely dilating a diseased vessel segment, which remains susceptible to postoperative diffuse restenosis.^4^ Contemporary conduit selection predominantly involves the left internal mammary artery, with supplementary use of saphenous vein grafts (SVGs) as needed, a strategy collectively termed single arterial grafting (SAG). However, accumulating angiographic evidence highlights the susceptibility of SVGs to progressive atherosclerosis and late occlusion,^5^ prompting growing interest in arterial-predominant revascularization strategies. In this context, multiple arterial grafting (MAG) and total arterial revascularization (TAR) have emerged as promising alternatives, warranting further investigation into their long-term clinical and survival benefits.

Despite a rapidly expanding body of literature consistently demonstrating superior clinical outcomes of MAG and TAR compared to conventional SAG,^6^^,^^7^ the adoption of these strategies remains paradoxically low. Recent reports indicate that MAG is utilized in <10% of CABG procedures, with TAR rates even lower.^8^ A potential explanation lies in the perceived technical challenges and reported longer operating times^9^ associated with arterial graft harvesting or grafting techniques.^10^ However, the lack of consensus on the optimal approach may have further hindered the widespread practice change within the surgical community.

The key distinction between MAG and TAR lies in their core strategic philosophy: MAG increases the number of arterial grafts, leveraging their superior long-term patency over venous conduits, whereas TAR eliminates SVGs entirely to mitigate their well-documented limitations. It is noteworthy that an increased use of arterial conduits also, on average, reduces the number of SVGs. The ongoing debate between these 2 arterial revascularization strategies has been perpetuated by a scarcity of literature, primarily due to their nonstandard implementation across the globe and subsequently limited patient samples.

This systematic review aims to comprehensively synthesize the existing evidence on the long-term survival and clinical outcomes of MAG and TAR compared to SAG in adults undergoing isolated CABG. Furthermore, the application of a network meta-analytical approach will enable an indirect comparison between MAG and TAR to assess their relative treatment effects.

## Methods

The reporting of this systematic review strictly followed the Preferred Reporting Items for Systematic Reviews and Meta-Analysis (PRISMA) guideline and was registered at the International Prospective Register of Systematic Reviews (PROSPERO) under CRD420251117898. Institutional approval was obtained from the Melbourne Health Human Research Ethics Committee (2025.089).

### Eligibility criteria

MAG was defined as a CABG technique involving 2 or more arterial conduits, with or without supplementary SVGs. TAR was defined as another technique exclusively utilizing arterial grafts to restore blood supply to compromised territories, regardless of the number of arterial conduits used. SAG, serving as the conventional control strategy, was defined as the use of a single arterial conduit combined with supplementary SVGs, typically considered equivalent to non-TAR in comparative studies evaluating TAR procedures. However, to allow for the indirect component of network meta-analysis, we only included non-TAR patient groups that were also SAG. No restriction was applied regarding the publication date. We considered studies eligible for inclusion if they:

1. Examined patients who have received isolated CABG;
2. Included a TAR or MAG patient group;
3. Included a non-TAR patient group that was also SAG;
4. Reported long-term patient survival, specifically HRs with CIs or Kaplan-Meier curves;
5. Were randomized controlled trials (RCTs) or observational studies with appropriate statistical adjustments, such as propensity-score methods or multivariable Cox regression; and
6. Had a sample size of each arm ≥100 after weighting/matching.

Exclusion criteria included non-English publications, case reports, conference abstracts or proceedings, and studies that reported revascularization with a single conduit or exclusively SVG. In studies with multiple comparative cohorts, the largest eligible groups were prioritized for quantitative synthesis. For overlapping data sets, the study with the largest sample size and longest follow-up was selected, unless separate publications reported distinct outcomes, in which case those outcomes were extracted from the relevant source. When a treatment cohort could be classified as both MAG and TAR, it was categorized as TAR to ensure greater accuracy, as TAR has been widely regarded in the literature as a subgroup strategy and a more precise description of MAG characterized by the exclusive use of arterial conduits.

### Outcome measures

The primary outcome was long-term all-cause mortality measured from the date of the index operation, chosen for its robustness as a definitive endpoint.

### Data source and search strategy

We systematically searched PubMed/Medical Literature Analysis and Retrieval System Online, Excerpta Medica Database, and the Cochrane Library from their inception through to the final search date of March 9, 2026. The search strategy included a comprehensive combination of controlled vocabulary (Medical Subject Headings terms), and keywords related to “coronary artery bypass”, “total arterial revascularization”, “multiple arterial grafting”, and “survival”. Complete search strategies are provided in Supplemental Table 1. In addition, references from relevant systematic reviews and included studies were cross-checked to identify potentially eligible studies. After deduplication, 3 authors (J.R., C.S., and W.Z.) formed the screening committee to independently conduct the screening process using web-based systematic review platform (Covidence; Veritas Health Innovation). Any disagreements were resolved through discussions within the screening committee. If consensus could not be reached, the final decision was made in consultation with the senior author (A.R.). Study selection involved initial screening of titles and abstracts, followed by full-text assessment against predefined eligibility and exclusion criteria. In cases of insufficient or unclear data, additional information was requested from corresponding authors via email or telephone.

### Data extraction

The following study characteristics were collected for comprehensive reporting: authors, year of publication, journal, study design, aims, adjustment methods, country, participating institutions, inclusion and exclusion criteria, completeness of follow-up, duration of follow-up, sample size, patient demographics, off-pump use, and exact graft configurations. The time-to-event outcomes were extracted as HRs and SEs. All extracted details were recorded in Excel (Microsoft Corp) with quality checks conducted before statistical analysis.

### Bias assessment

We assessed the risk of bias in RCTs using the Cochrane Risk-of-Bias Tool 2,^11^ evaluating 5 key domains: bias arising from the randomization process; deviations from intended interventions; missing outcome data; measurement of outcomes; and selection of the reported results. For nonrandomized observational studies, we used the Risk Of Bias In Non-randomized Studies of Interventions-I tool,^12^ a validated seven-domain framework assessing biases from confounding, participant selection, classification of interventions, deviations from intended interventions, missing data, measurement of outcomes, and selective reporting. The overall certainty of evidence was evaluated using the Grading of Recommendations Assessment, Development, and Evaluation (GRADE) methodology. Risk-of-bias assessment and quality appraisal were performed independently by 2 reviewers (J.R. and C.S.), with discrepancies resolved through consultation with a third investigator (D.H.T.).

### Statistical analysis

Continuous variables were summarized as mean ± SD or median (IQR), depending on data distribution. Where necessary, a validated conversion tool was utilized to estimate means from median values.^13^ Categorical variables were summarized as counts with percentages.

A Bayesian network meta-analysis (B-NMA) was performed to synthesize direct and indirect evidence across eligible studies comparing CABG strategies. Because HRs derived from Cox proportional hazards models are approximately normally distributed on the logarithmic scale, reported HRs were transformed to log-HRs before analysis. Corresponding SEs were derived from the reported 95% CIs when necessary. A contrast-based random-effects network meta-analysis was then fitted using a normal likelihood with an identity link, with study-specific treatment effects weighted by the inverse variance of the log-HR. This framework is appropriate for time-to-event outcomes expressed as relative treatment effects under the proportional hazards assumption. Pooled treatment effects were subsequently exponentiated and reported as HRs with 95% credible intervals (CrIs).

The B-NMA was conducted in R (R Core Team) using the gemtc package, which implements Markov Chain Monte Carlo (MCMC) simulation through Just Another Gibbs Sampler. Vague (minimally informative) prior distributions were specified so that the posterior estimates were driven by the observed data rather than by external assumptions. Following the default package specification, the basic treatment-effect parameters were assigned normal priors centered at zero with a SD more than an order of magnitude larger than the range of plausible log-HRs, so that the prior was effectively flat across all clinically credible effects. The common between-study heterogeneity SD was assigned a uniform prior bounded below at zero and extending well beyond the largest heterogeneity compatible with the data. Convergence was confirmed by the Brooks-Gelman-Rubin diagnostic (all potential scale reduction factors = 1.00) and inspection of trace plots. Six parallel Markov chains were run, each with 10,000 burn-in iterations followed by 100,000 sampling iterations, with a thinning interval of 10 to reduce autocorrelation.

Network geometry was illustrated using network plots, in which nodes represented treatment strategies and edges represented available direct comparisons. Relative treatment effects were summarized using forest plots with SAG as the reference comparator. In addition to comparisons vs SAG, TAR was compared directly with MAG within the network framework.

Treatment ranking was estimated using rank probabilities and the surface under the cumulative ranking curve (SUCRA), where values closer to 1 indicate a higher probability of being the most effective strategy. The stability of the SUCRA-based treatment ranking was assessed using a leave-one-out influence analysis, in which each study was omitted in turn and the ranking recomputed, with agreement between the full and reduced rankings quantified using Cohen kappa.^14^

Because a random-effects network meta-analysis of HRs estimates a single common between-study SD shared across all comparisons, between-study heterogeneity was quantified by this parameter, τ, together with its variance, τ^2^. This parameter is estimated jointly from all direct and indirect evidence and therefore reflects heterogeneity across the entire network rather than within any single pairwise contrast, and it was summarized using its posterior estimate and 95% CrI. Because I^2^ is intrinsically a pairwise measure that does not generalize to a multicomparison network, it was not used to quantify heterogeneity in the network meta-analysis. Both τ and τ^2^ were therefore treated as the definitive measures of between-study heterogeneity in this framework.

The assumptions of transitivity and consistency were considered a priori. Transitivity was supported by restricting inclusion to risk-adjusted comparative studies evaluating clinically comparable populations, interventions, and outcomes. Consistency between direct and indirect evidence was assessed locally using node-splitting analyses.^15^ Globally, inconsistency across the entire network was further assessed using the design-by-treatment interaction model^16^ through its between-designs Q statistic.

### Individual patient data reconstruction

For all studies reporting Kaplan-Meier visualizations, individual patient data (IPD) were reconstructed by digitizing survival curves and applying a validated iterative algorithm proposed by Guyot et al,^17^ assuming constant and noninformative censoring. To preserve the covariate adjustment applied in the primary studies, the reconstruction algorithm was applied exclusively to adjusted or matched survival curves with balanced covariate distributions and never to crude curves.

### Sensitivity analyses

To assess the robustness of the network findings, conventional pairwise meta-analyses were performed on the included studies using both fixed-effect and random-effects models. Reported HRs and 95% CIs were transformed to the log-HR scale, with SEs derived from the reported confidence limits using the normal approximation. Estimates were pooled by generic inverse-variance weighting in the meta package. Fixed-effect (common-effect) models used the inverse-variance method, and random-effects models estimated the between-study variance by restricted maximum likelihood. Heterogeneity was summarized with the I^2^ statistic, with values of approximately 25%, 50%, and 75% classified as low, moderate, and high heterogeneity, respectively.^18^ Forest plots were generated to display individual study estimates and pooled effects.

In the second sensitivity analysis, the Bayesian random-effects network meta-analysis was repeated after excluding all studies whose HRs were derived from IPD reconstruction, thereby restricting the network to studies reporting adjusted HRs directly. The model specification, prior distributions, and MCMC settings were identical to those of the primary analysis, and pooled effects were again summarized as HRs with 95% CrIs to determine whether the inclusion of reconstructed data materially influenced the network estimates.

## Results

**Figure 1:**
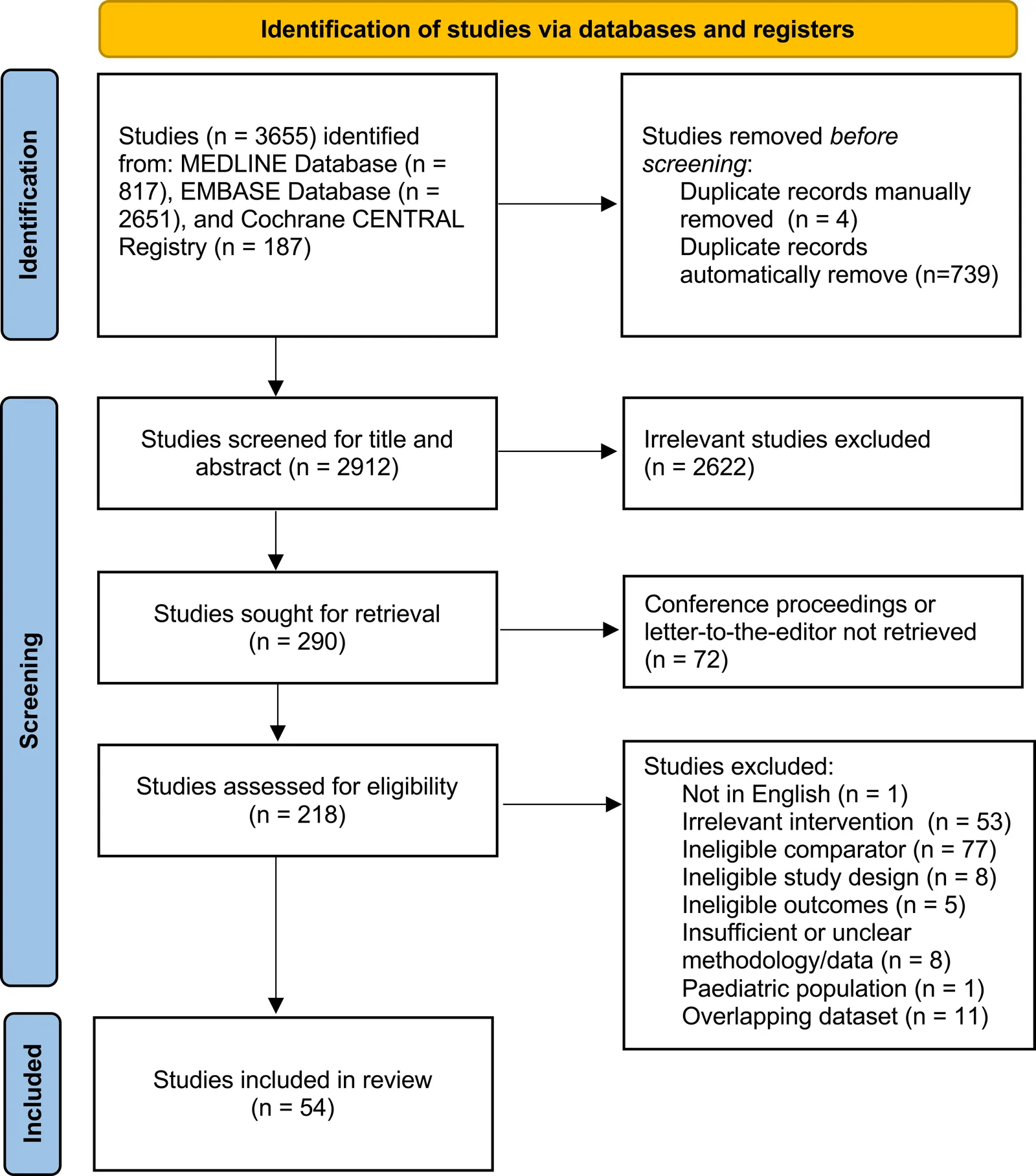
Preferred Reporting Items for Systematic Reviews and Meta-Analyses Flow Diagram of Study Selection Flow diagram^72^ summarizing the identification, screening, eligibility assessment, and inclusion of studies in the systematic review. CENTRAL = Cochrane Central Register of Controlled Trials; EMBASE = Excerpta Medica Database; MEDLINE = Medical Literature Analysis and Retrieval System Online.

The systematic literature search identified 3,655 records, including 817 from Medical Literature Analysis and Retrieval System Online, 2,651 from Excerpta Medica Database, and 187 from the Cochrane Central Register of Controlled Trials. After removal of 743 duplicate records (739 identified automatically and 4 manually), 2,912 records remained for title and abstract screening. Of these, 2,622 were excluded for not meeting the inclusion criteria, leaving 290 articles for full-text review. Following full-text assessment, 218 studies were evaluated for eligibility. A total of 164 studies were excluded for the following reasons: irrelevant intervention (n = 53), ineligible comparator (n = 77), ineligible study design (n = 8), ineligible outcomes (n = 5), insufficient or unclear methodological or outcome data (n = 8), non-English language (n = 1), pediatric population (n = 1), and overlapping data sets (n = 11). A total of 54 eligible investigations^8^^,^^19^, ^20^, ^21^, ^22^, ^23^, ^24^, ^25^, ^26^, ^27^, ^28^, ^29^, ^30^, ^31^, ^32^, ^33^, ^34^, ^35^, ^36^, ^37^, ^38^, ^39^, ^40^, ^41^, ^42^, ^43^, ^44^, ^45^, ^46^, ^47^, ^48^, ^49^, ^50^, ^51^, ^52^, ^53^, ^54^, ^55^, ^56^, ^57^, ^58^, ^59^, ^60^, ^61^, ^62^, ^63^, ^64^, ^65^, ^66^, ^67^, ^68^, ^69^, ^70^, ^71^ reporting all-cause mortality were included in the systematic review (Figure 1).

### Study characteristics

The included comparative investigations, published between 2004 and 2026, comprised a total of 560,723 patients. Most studies were retrospective observational cohorts, with 1 RCT and 3 post hoc analyses identified (Table 1). Seven studies^29^^,^^40^^,^^41^^,^^56^^,^^60^^,^^64^^,^^69^ did not directly report HRs and therefore required IPD reconstruction from Kaplan-Meier survival curves to estimate HRs using established Guyot methods (Supplemental Table 2). Although a minimum threshold of 100 patients per treatment arm after matching or weighting was prespecified in our protocol, no otherwise-eligible study was excluded on this basis. This criterion was therefore nonbinding and did not affect the composition of the included evidence.

**Table 1:** Characteristics of Studies Included in Bayesian Network Meta-Analysis

First Author (Year)	Nation	N	Study Type	Mean Follow-Up	Adjustment Methods	Patients
MAG vs SAG Investigations						
Aboul-Hassan (2024)^19^	Poland	14,566	Retrospective	Median 5.7 (IQR: 3.4-7.9)	PSM	Males
Barili^21^	Italy	20,870	Retrospective	Median 7.9	IPTW	General
Carrier^24^	Canada	6,655	Retrospective	NA	Multivariable Cox	General
Chaubey^25^	United Kingdom	528	Retrospective	Median 15.5 (IQR: 9-21)	Multivariable Cox	Age <60 y, elective
Chikwe^26^	United States	7,176	Retrospective	Median 7.8 (IQR: 5-10)	PSM	Multivessel disease
Gaudino^30^	United States	19,024	Retrospective	NA	PSM	Multivessel disease
Gikandi^31^	United States	25,969	Retrospective	NA	Multivariable Cox	On-pump
Guru^33^	Canada	10,982	Retrospective	NA	PSM	General
Habib^34^	United States	2,832	Retrospective	NA	PSM	Male
Hachiro^35^	Japan	1,221	Retrospective	6.7	IPTW	Off-pump
Iribarne^36^	United States	3,494	Retrospective	Median 13.2 (IQR: 7.4-17.7)	PSM	General
Jameie^37^	Iran	23,798	Retrospective	NA	IPTW	General
Janiec^39^	Sweden	47,379	Retrospective	9.3 ± 4.2	Multivariable Cox	General
Joo^40^	South Korea	732	Retrospective	7.0 ± 2.0	PSM	General
Kieser^38^	Canada	5,067	Retrospective	13.9	Multivariable Cox	General
Kurlansky^41^	United States	2,452	Retrospective	NA	PSM	Multivessel disease
Lin^43^	United States	520	Retrospective	12	PSM	General
Locker^44^	United States	1,602	Retrospective	7.9 ± 4.7	PSM	Multivessel disease
Pu^45^	Canada	10,931	Retrospective	NA	PS adjustment	LMD
Rocha^49^	Canada	17,258	Retrospective	4.2	PSM	General
Rosenblum^50^	United States	16,692	Retrospective	NA	IPTW	General
Samadashvili^51^	United States	21,656	Retrospective	Median 6.5	PSM	Multivessel disease
Schwann^52^	United States	10,460	Retrospective	8.7 ± 4.4	Multivariable Cox	General
Thuijs^53^	Multinational	1,466	Post hoc	12.6	Multivariable Cox	Multivessel disease or LMD
Toumpoulis^54^	United States	980	Retrospective	4.7 ± 3.0	PSM	Diabetics
Tranbaugh^55^	United States	11,650	Retrospective	NA	Multivariable Cox	General
Yamaguchi^56^	Japan	1,154	Retrospective	8.6	PSM	Nondiabetics
Zhang^62^	China	919	Retrospective	NA	PSM	General
Zhu^63^	China	378	Retrospective	6.9	PSM	General
Benedetto^58^	United Kingdom	4,014	Retrospective	Median 7.5 (IQR: 3.6-11.3)	PSM	General
Gatti^59^	Italy	494	Retrospective	NA	PSM	Females
Popovic^61^	France	260	Retrospective	6.2 ± 3.8	PSM	Reduced LVEF
Sabik^8^	United States	200,808	Retrospective	Median 5.3	IPTW	General
Dayan^65^	Uruguay	5,522	Retrospective	Median 7.8 (IQR: 3.9-12.2)	PSM	General
Al-Holy^64^	United Kingdom	1,324	Retrospective	NA	PSM	Diabetics
TAR vs SAG investigations						
Bisleri^22^	Italy	350	Retrospective	NA	PSM	General
Browne^23^	Multinational	1,019	Post hoc	4.8	PS adjustment	General
Hamilton^71^	Australia	225	RCT	15	Randomized	General
DiBacco^27^	Italy	718	Retrospective	Median 8.4 (range 0.9-15.4)	PSM	Diabetics
Dimitrova^28^	United States	566	Retrospective	NA	PSM	Women
Garatti^29^	Italy	452	Retrospective	13.8 ± 3.6	PSM	Multivessel disease, on-pump
Goldstone^32^	United States	11,626	Retrospective	NA	PSM	Multivessel disease
Legare^42^	Canada	4,696	Retrospective	NA	Multivariable Cox	On-pump
Qureshi^46^	United Kingdom	2,374	Retrospective	NA	PSM	General
Raja^47^	United Kingdom	1,386	Retrospective	NA	PSM	Off-pump
Zacharias^57^	United States	4,200	Retrospective	12	PSM	General
Jeong^60^	South Korea	584	Retrospective	NA	PSM	Off-pump
Medalion^68^	Israel	1,627	Retrospective	8.17 ± 4.45	PS adjustment	Elderly (≥70 years)
Grau^66^	United States	1,856	Retrospective	NA	PSM	NA
Kunihara^67^	Japan	208	Retrospective	NA	PSM	Diabetic, multivessel disease, on-pump
Taggart^70^	Multinational	1,927	Post hoc	5.2	IPTW	General
TAR vs MAG investigations						
Aboul-Hassan^20^	Poland	462	Retrospective	Median 7.43 (IQR: 4.4-12.3)	PSM	General
Ren^48^	Australia	22,274	Retrospective	Median 5.0	PSM	General
Navia^69^	Argentina	3,340	Retrospective	Median 5.5	Multivariable Cox	Multivessel disease

IPTW = inverse probability of treatment weighting; LMD = left main disease; LVEF = left ventricular ejection fraction; MAG = multiple arterial grafting; NA = not available; PS = propensity score; PSM = propensity score matching; RCT = randomized controlled trial; SAG = single arterial grafting; TAR = total arterial revascularization.

All studies used statistical adjustment techniques to address baseline confounding. Propensity score matching was the most common method (34 studies, 63.0%), followed by multivariable Cox regression (10 studies, 18.5%), inverse probability of treatment weighting (6 studies, 11.1%), and propensity score adjustment (3 studies, 5.6%). One study (1.9%) was an RCT. Baseline demographic and clinical characteristics of patients in the intervention and control groups are presented in Supplemental Tables 3 and 4, respectively.

### Bayesian hierarchical network meta-analysis of all-cause mortality

**Figure 2:**
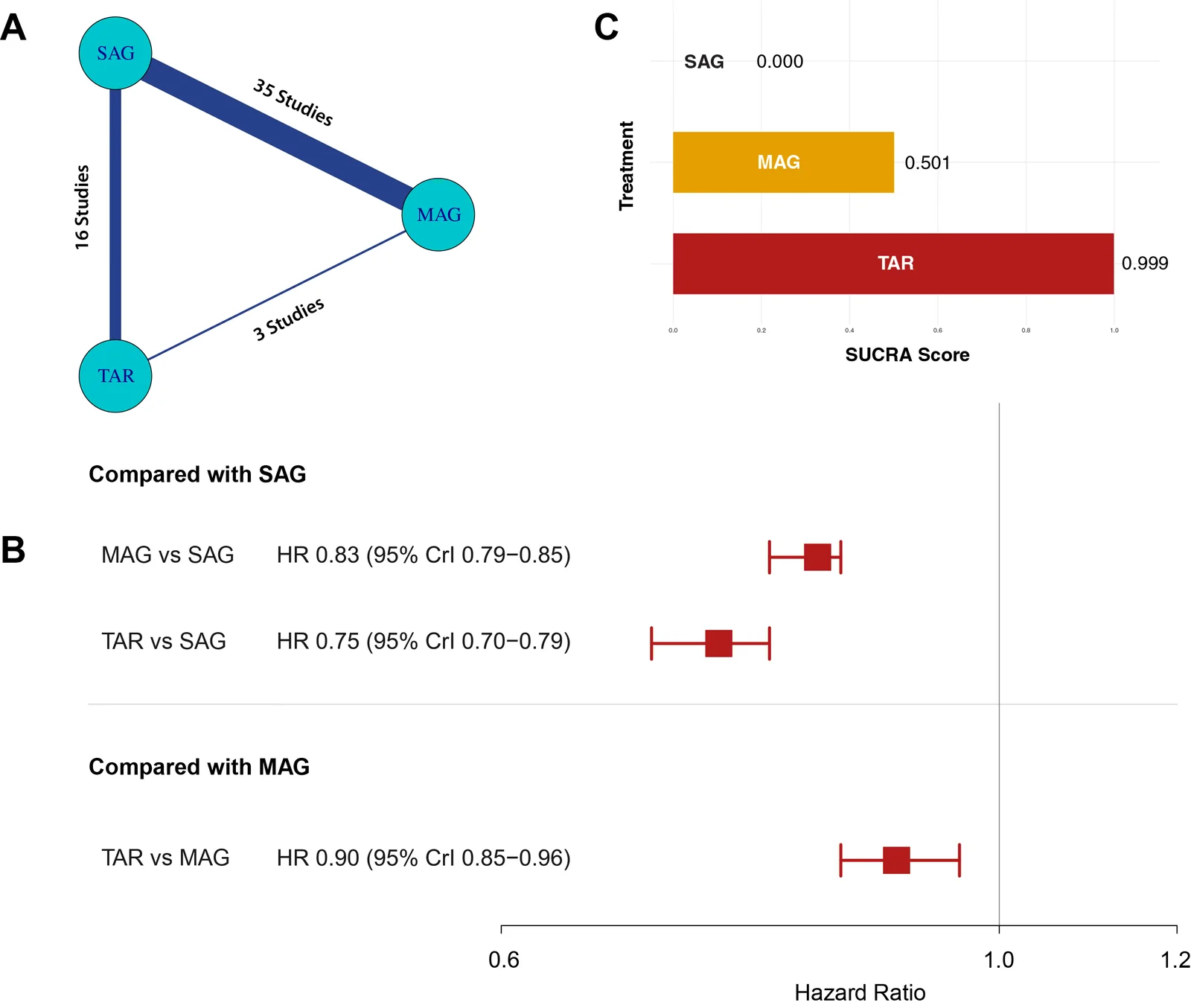
Network Analysis of All-Cause Mortality for Surgical Revascularization Strategies (A) Network of direct comparisons among SAG, MAG, and TAR. Node size reflects the number of studies evaluating each strategy, and line thickness represents the number of direct comparisons between treatments. (B) Forest plot showing HRs from the Bayesian network meta-analysis comparing long-term all-cause mortality between revascularization strategies. Points represent posterior mean HRs and horizontal lines represent 95% credible intervals. (C) Ranking of treatment strategies according to the surface under the cumulative ranking curve (SUCRA). Higher SUCRA values indicate a greater probability of being the most effective treatment. CrI = credible interval; MAG = multiple arterial grafting; SAG = single arterial grafting; TAR = total arterial revascularization.

The evidence network comprised 3 CABG treatment strategies: SAG, MAG, and TAR. The MAG-SAG comparison was the most frequently documented (35 studies), followed by TAR-SAG (16 studies), whereas only 3 studies directly compared TAR against MAG. The network was therefore predominantly anchored by SAG comparisons, with indirect evidence contributing substantially to the TAR-versus-MAG estimate (Figure 2A). This estimate should accordingly be interpreted as predominantly indirect, with correspondingly wider CrIs than for directly anchored comparisons.

In the B-NMA, both TAR and MAG were associated with significantly lower risk of long-term all-cause mortality compared with SAG. Compared with SAG, MAG was associated with a 17% lower risk of death (HR: 0.83; 95% CrI: 0.79-0.85). TAR demonstrated a greater survival improvement, corresponding to a 25% lower risk of death compared with SAG (HR: 0.75; 95% CrI: 0.70-0.79). When TAR was compared directly with MAG, TAR was associated with a modest but statistically significant reduction in mortality (HR: 0.90; 95% CrI: 0.85-0.96), indicating an incremental survival benefit beyond that achieved with MAG alone (Figure 2B). Between-study heterogeneity was low, with a posterior mean between-study SD τ of 0.0485 (95% CrI: 0.012-0.089) and a corresponding between-study variance τ^2^ of 0.002, indicating minimal variability in treatment effects beyond sampling error.

Treatment ranking based on the SUCRA suggested a ranking consistent with the effect estimates. TAR had the highest probability of ranking most favorably for survival, with a SUCRA value of 0.999. MAG ranked intermediate with a SUCRA of 0.501, whereas SAG consistently ranked lowest with a SUCRA close to 0 (Figure 2C). The treatment ranking was robust to the exclusion of individual studies, with perfect agreement between the full-network ranking and every leave-one-out ranking (Cohen kappa = 1 for all studies). Together, these findings suggest a stepwise survival advantage associated with increasing use of arterial conduits, with TAR demonstrating the most favorable long-term survival outcomes, followed by MAG, and SAG associated with the least favorable outcomes.

### Model convergence and consistency diagnostics

The Brooks-Gelman-Rubin diagnostic confirmed convergence, with all potential scale reduction factors equal to 1.00 and a multivariate potential scale reduction factor of 1.00. The convergence of the B-NMA was also assessed using trace and posterior density plots of the MCMC simulations, which demonstrated adequate mixing and stationarity across chains (Supplemental Figure 1). Monte Carlo errors were negligible relative to posterior SDs (all <1%), indicating stable parameter estimation. Model fit was evaluated using the posterior mean residual deviance, which was 63.7 for 54 data points, yielding a deviance-to-data-point ratio of 1.18, consistent with an appropriate level of agreement between model predictions and the observed data. Node-splitting assessment did not detect statistically significant inconsistency between direct and indirect evidence across the network comparisons for all-cause mortality (Supplemental Figure 2). Global assessment using the design-by-treatment interaction model further reported no statistically significant inconsistency between direct and indirect evidence (Q = 3.18; *P* = 0.07).

### Aggregated Kaplan-Meier survival curve

**Figure 3:**
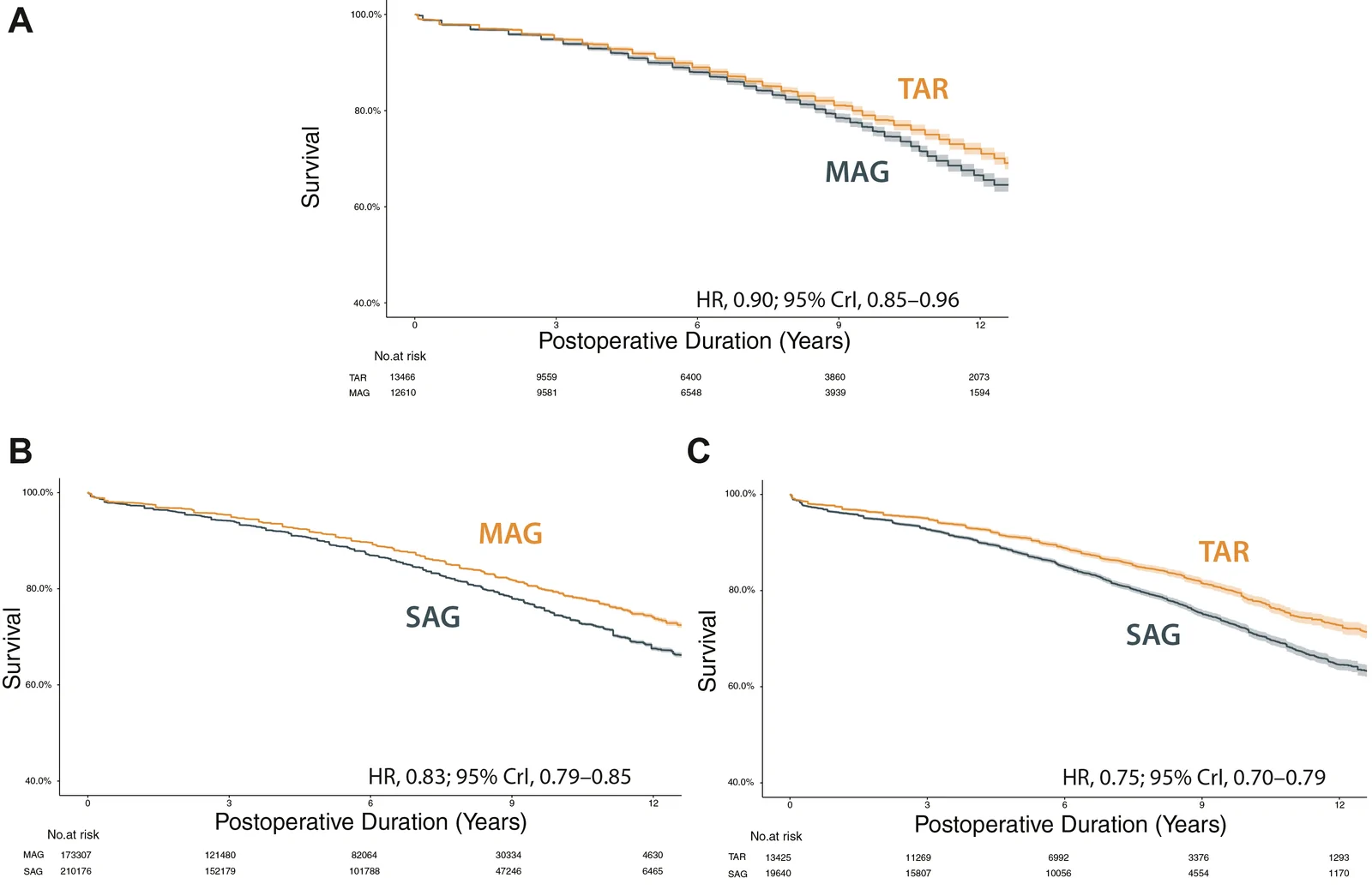
Aggregated Kaplan-Meier Long-Term Survival Curves Using Reconstructed Individual Patient Data Pairwise comparisons are presented for (A) TAR vs MAG, (B) MAG vs SAG, (C) and TAR vs SAG. The aggregated Kaplan-Meier estimates were generated as part of the meta-analysis using reconstructed individual patient data derived from published adjusted or matched survival curves with Guyot algorithm.^17^ Numbers at risk are shown below each panel at the corresponding time points. HRs with 95% credible intervals are displayed for each comparison. Abbreviations as in Figure 2.

Reconstructed individual patient-level survival data were pooled across included studies to generate Kaplan-Meier curves for each pairwise comparison (Figure 3). Consistent with the meta-analytical findings, TAR was associated with superior long-term survival over both MAG and SAG, and MAG conferred a survival advantage over SAG. The curves separated progressively from approximately 3 years postoperatively and remained divergent throughout follow-up, supporting a sustained late survival benefit of arterial revascularization.

### Risk of bias and quality assessment

Overall, the methodological quality of the included studies was generally high. The majority of studies were judged to have a low-moderate risk of bias across most domains from the Risk Of Bias In Non-randomized Studies of Interventions-I, reflecting the use of large clinical registries, rigorous adjustment methods, and adequate follow-up for mortality outcomes. Only 15 studies were assessed as having a high risk of bias, primarily due to limitations in adjustment for baseline confounders, incomplete reporting of follow-up completeness, or potential selection bias inherent to retrospective study designs. No studies were excluded on the basis of methodological quality. The only RCT was assessed with Cochrane Risk-of-Bias Tool 2 with a low risk of bias overall (Supplemental Figure 3).

The certainty of evidence was assessed using the GRADE framework adapted for network meta-analysis. For the primary outcome of long-term all-cause mortality, the certainty of evidence was rated as moderate for MAG vs SAG and TAR vs SAG, reflecting consistent effect estimates, low statistical heterogeneity, and precise treatment effects despite the predominance of adjusted observational studies. In contrast, the certainty of evidence for TAR vs MAG was rated as low, because this comparison was informed by only limited direct evidence and relied substantially on indirect estimation within the network (Supplemental Table 5).

### Sensitivity analyses

**Figure 4:**
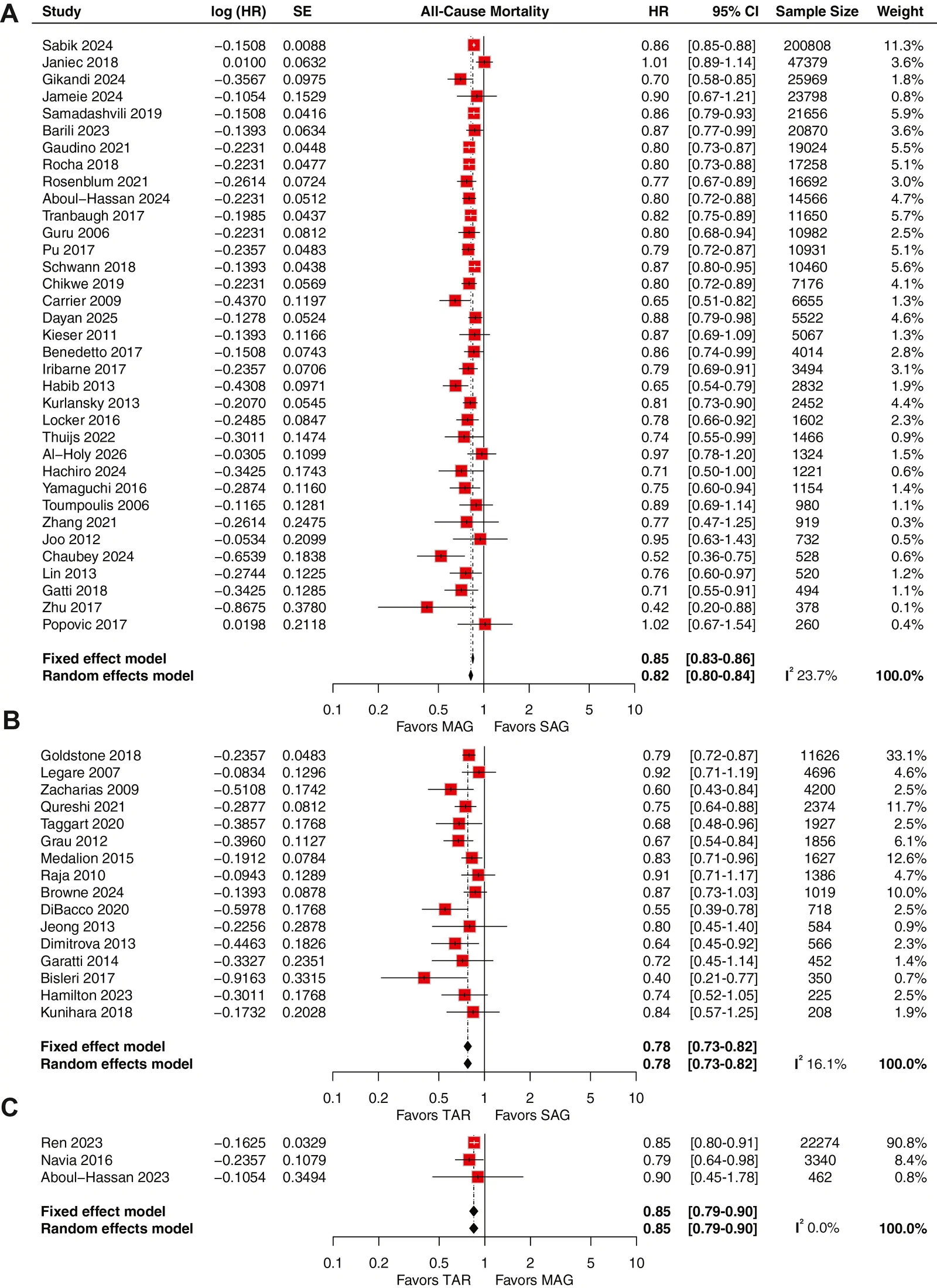
Sensitivity Analyses using Conventional Pairwise Meta-Analyses of Long-Term Mortality Forest plots showing pooled HRs from conventional inverse-variance weighted pairwise meta-analyses comparing (A) MAG vs SAG, (B) TAR vs SAG, and (C) TAR vs MAG. HRs and 95% CIs were calculated on the logarithmic scale and pooled using both fixed-effect and random-effects models. Squares represent study-specific estimates and horizontal lines indicate 95% CIs; the size of the squares reflects study weighting. Diamonds represent pooled estimates, with widths corresponding to the 95% CI. The vertical line denotes the line of no effect. Values < 1 indicate a survival benefit favoring MAG or TAR, depending on the comparison shown. Abbreviations as in Figure 2.

Our conventional pairwise meta-analysis demonstrated a consistent survival benefit associated with MAG compared with SAG with random-effects reporting pooled HR of 0.82 (95% CI: 0.80-0.84), whereas the fixed-effects model produced a similar estimate of 0.85 (95% CI: 0.83-0.86), indicating a consistent reduction in long-term mortality with MAG (Figure 4A). For the comparison between TAR and SAG, pooled estimates also favored TAR (Figure 4B). The random-effects model showed a pooled HR of 0.78 (95% CI: 0.73-0.82), with similar results from the fixed-effect model. Comparisons between TAR and MAG were limited to 3 studies (Figure 4C), but pooled analysis suggested a modest additional survival advantage with TAR. Both random- and fixed-effect estimates showed an HR of 0.85 (95% CI: 0.79-0.90) favoring TAR over MAG. All heterogeneities from sensitivity analyses were low (Figure 4).

In another sensitivity analysis restricted to studies reporting HRs directly, excluding all studies requiring IPD reconstruction, the treatment effects were consistent with the primary investigation. Relative to SAG, both MAG (HR: 0.83; 95% CrI: 0.79-0.85) and TAR (HR: 0.75; 95% CrI: 0.70-0.79) were associated with a lower hazard, and TAR also showed a lower hazard than MAG (HR: 0.91; 95% CrI: 0.86-0.98). The stability of these estimates supports the robustness of our findings to the inclusion of reconstructed data.

## Discussion

In this systematic review and Bayesian hierarchical network meta-analysis including approximately 560,000 patients undergoing CABG, we observed a consistent and incremental survival benefit associated with increasing use of arterial conduits (Central Illustration). Both MAG and TAR were associated with significantly improved long-term survival compared with conventional SAG. Importantly, the network meta-analytic framework permitted coherent integration of direct and indirect evidence to estimate the TAR-versus-MAG contrast, a comparison previously inaccessible to conventional pairwise synthesis owing to the scarcity of direct head-to-head studies, to demonstrate that TAR was associated with a modest but statistically significant survival advantage over MAG. These findings suggest a hierarchical pattern of treatment effectiveness, whereby complete arterial revascularization appears to be associated with the greatest survival benefit, followed by MAG, with SAG associated with comparatively less favorable long-term outcomes. Treatment ranking analyses using SUCRA supported this gradient, with TAR demonstrating the highest probability of being the most effective grafting strategy. Reconstructed Kaplan-Meier survival curves and sensitivity analyses were all consistent with these findings.

**Central Illustration:**
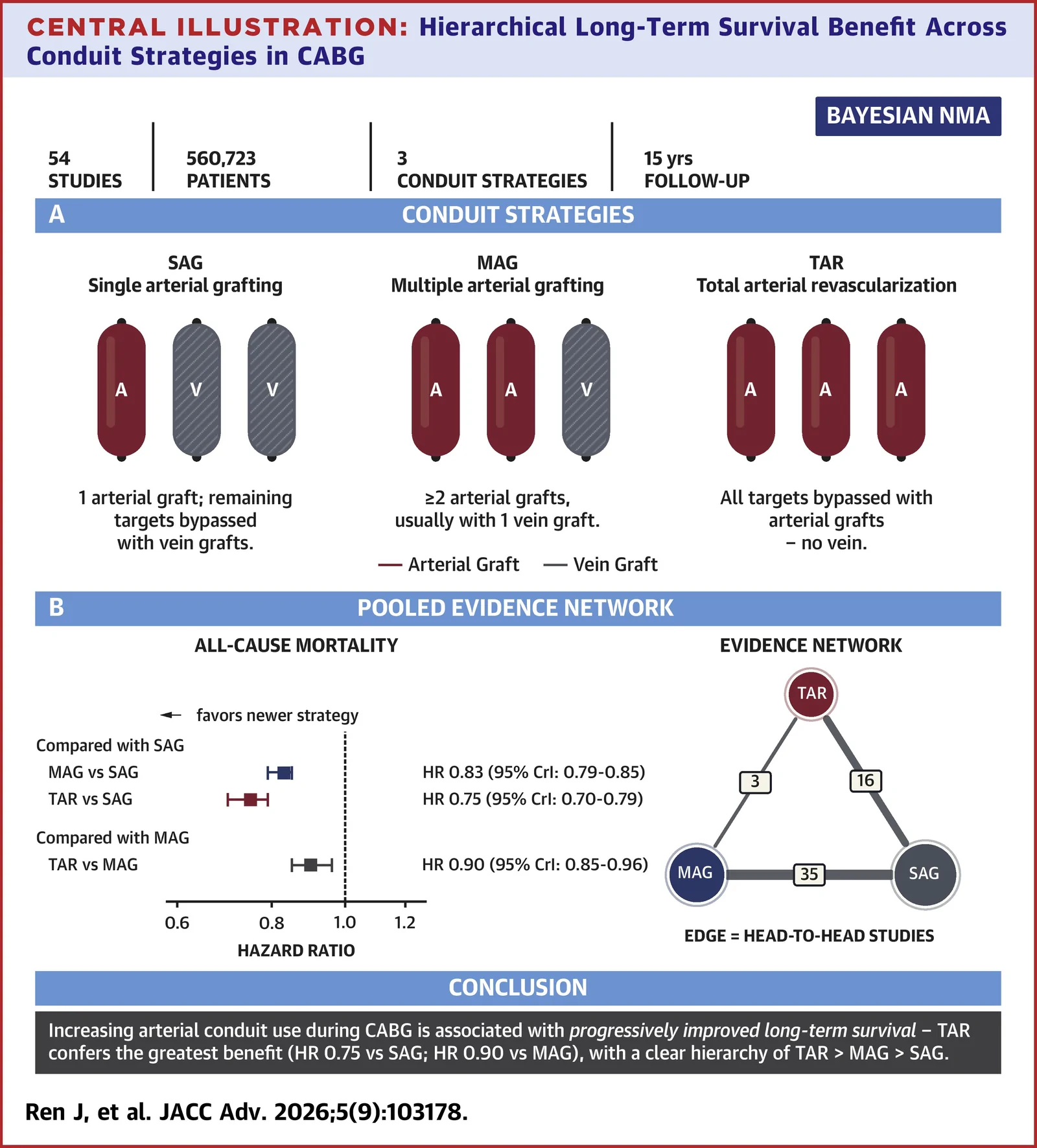
Hierarchical Long-Term Survival Benefit Across Conduit Strategies in CABG Bayesian network meta-analysis of 54 studies (n = 560,723; follow-up to 15 years) comparing single arterial grafting (SAG), multiple arterial grafting (MAG), and total arterial revascularization (TAR). (A) The 3 conduit strategies; arterial grafts in red, saphenous vein grafts in gray. (B) Pooled adjusted HRs (95% credible interval [CrI]) for all-cause mortality and the evidence network. A consistent hierarchy was observed (TAR > MAG > SAG). CABG = coronary artery bypass grafting; NMA = network meta-analysis.

The survival advantage associated with arterial grafting strategies is biologically plausible and consistent with long-standing observations regarding graft durability.^5^ Arterial conduits, particularly the internal mammary and radial arteries, demonstrate superior long-term patency compared with SVGs due to their intrinsic long-term resistance to atherosclerosis, favorable endothelial function, and greater adaptability to coronary arterial hemodynamics.^73^^,^^74^ In contrast, SVGs are susceptible to progressive intimal hyperplasia, accelerated atherosclerosis, and late occlusion, with angiographic studies demonstrating substantial attrition within the first decade after surgery.^75^^,^^76^ Within this mechanistic framework, MAG increases the number of arterial conduits available for revascularization, thereby reducing dependence on venous grafts. TAR extends this principle further by eliminating SVGs entirely. The further survival gain seen with TAR relative to MAG in the present analysis therefore supports the hypothesis that complete avoidance of venous conduits may translate into additional postoperative clinical benefit. Graft patency was not consistently reported across the included studies and was therefore not synthesized in the present meta-analysis. The mechanistic links between superior arterial conduit patency and improved long-term survival are accordingly framed here as biologically plausible hypotheses supported by ancillary angiographic literature, rather than as conclusions of the present analysis.

A critical consideration when interpreting the relative effects between TAR and MAG is the inherent overlap in their classification. By definition, MAG encompasses any CABG procedure using 2 or more arterial conduits, irrespective of whether supplementary SVGs are also used. Consequently, MAG cohorts inevitably contain an unquantifiable subset of patients who received exclusively arterial grafts and therefore met the criteria for TAR. Because individual studies did not consistently differentiate between these 2 categories, this classification imprecision has 2 important analytical implications. First, the inclusion of TAR patients within MAG cohorts may have inflated the observed survival benefit of MAG relative to SAG, as outcomes attributable to complete arterial revascularization would have been partially attributed to the broader MAG category. Second, the estimated survival advantage of TAR over MAG is likely conservative, as comparing TAR against a MAG group already containing some TAR patients dilutes the contrast and biases the indirect comparison toward the null. The true added benefit of TAR may therefore exceed the HR of 0.90 (95% CrI: 0.85-0.96) reported here. This misclassification bias underscores the need for future studies to explicitly distinguish between MAG with and without supplementary SVGs when reporting outcomes.

Simultaneously, our systematic literature search found that direct comparisons between TAR and MAG remain scarce, largely reflecting the persistently low utilization of complete arterial techniques in contemporary surgical practice. By integrating both direct and indirect evidence within a network meta-analytic framework, the present study provides one of the most comprehensive comparative evaluations of these strategies to date. The results reinforce the concept that arterial grafting strategies exist along a continuum of benefit, with TAR representing the most extensive implementation of arterial revascularization principles. Large registry analyses indicate that MAG is used in only a minority of CABG procedures worldwide, typically ranging between approximately 10% to 15% of cases,^77^^,^^78^ with the use of TAR almost certainly lower. The reasons for this disparity are likely multifactorial. Although the technical principles of MAG or TAR do not fundamentally differ from conventional CABG–often requiring only substitution of a venous conduit with an additional arterial graft such as the radial artery or right internal mammary artery–the perceived operative complexity, concerns regarding conduit harvest, and longer operative times may discourage routine implementation.^79^ The adoption of advanced arterial grafting strategies is also strongly influenced by surgeon experience and institutional practice patterns, with studies demonstrating substantial variation attributable to surgeon- and hospital-level factors rather than patient characteristics alone.^80^^,^^81^

In this context, the future expansion of arterial revascularization strategies may depend less on technical feasibility and more on the development of structured training pathways and institutional expertise in advanced conduit configurations. Establishing systematic training in MAG or TAR during cardiothoracic surgical training, along with institutional protocols promoting arterial graft use, may be necessary to translate the growing body of evidence into routine clinical practice. Given the consistent evidence of improved long-term survival associated with arterial revascularization, including across diverse patient populations such as individuals with diabetes, advanced age, or female sex, broader adoption of these strategies may represent an important opportunity to improve long-term outcomes following coronary artery bypass surgery.^28^^,^^82^^,^^83^

These findings also underscore the importance of clearer reporting and classification of arterial revascularization strategies in future studies. Although MAG and TAR are often discussed interchangeably within the broader concept of arterial revascularization, they represent distinct surgical philosophies and conduit configurations. Explicit differentiation between these approaches would improve comparability across studies and help clarify the incremental benefits associated with complete arterial revascularization. In this context, TAR may be more appropriately regarded as a distinct subset of arterial revascularization strategies, representing the most comprehensive application of arterial grafting principles and potentially offering the greatest long-term durability and survival benefit. Future randomized evidence^84^^,^^85^ would provide valuable insights into whether the observed advantages arise primarily from avoiding SVG failure or from the additive benefits of increasing arterial conduit use.

### Study Limitations

Several limitations warrant consideration. First, although all included studies applied rigorous within-study adjustment for baseline confounding using propensity score matching, inverse probability of treatment weighting, or multivariable Cox regression, and only adjusted HRs were pooled, residual confounding from unmeasured factors such as frailty, intraoperative findings, and surgeon judgment regarding patient suitability for complex arterial revascularization cannot be entirely excluded. This limitation was compounded by the absence of IPD, because pooling relied on aggregate published estimates. The lack of IPD precluded covariate adjustment and subgroup analyses across the network, and it also constrained assessment of the transitivity assumption, since the included studies did not report a standardized set of baseline covariates and the available effect modifiers differed substantially between studies. Definitive validation will therefore require a harmonized IPD meta-analysis or large pragmatic trials. Second, only 3 studies provided direct head-to-head comparisons between TAR and MAG, with the remainder of the contrast derived from indirect estimation anchored through SAG. Although indirect estimation is a recognized and methodologically valid feature of NMA, the CrI for the TAR-versus-MAG estimate should be interpreted accordingly. The large aggregate sample and the Bayesian framework nevertheless enabled coherent integration of direct and indirect evidence to address this clinically important comparison. Third, TAR has been perceived as a technically demanding procedure disproportionately performed by experienced, high-volume surgeons at centers with established arterial revascularization programs. Surgeon case volume, learning-curve effects, and center-level expertise were not consistently reported across the included studies and could not be incorporated as covariates. Prospective randomized data will ultimately be required to isolate the conduit effect from operator and institutional factors. Finally, nonfatal endpoints such as major adverse cardiac and cerebrovascular events, repeat revascularization, and graft patency were not synthesized because outcome definitions and ascertainment intervals varied substantially across studies and reporting was incomplete. All-cause mortality was therefore selected as the most objective and reliably captured endpoint.

## Conclusions

In this large systematic review and B-NMA, increasing the use of arterial conduits during CABG was associated with progressively improved long-term survival. Both MAG and TAR demonstrated significant survival advantages compared with conventional SAG, with TAR showing an additional incremental benefit relative to MAG. These findings support the concept that arterial-dominant revascularization strategies may offer superior long-term durability and favor broader use of arterial grafting in contemporary coronary surgery. However, as the TAR-versus-MAG comparison was supported by predominantly indirect network evidence (GRADE low certainty), this incremental advantage also warrants confirmation in prospective randomized trials.

Perspective **COMPETENCY IN MEDICAL KNOWLEDGE:** In a Bayesian network meta-analysis of 54 studies of isolated CABG, long-term survival improved stepwise with greater arterial conduit use. Multiple arterial grafting conferred a survival advantage over single arterial grafting, and total arterial revascularization provided the greatest benefit.

**TRANSLATIONAL OUTLOOK:** Because the comparison of total arterial revascularization with multiple arterial grafting relied largely on indirect evidence, randomized trials are needed to confirm the incremental benefit of eliminating vein grafts and to support broader adoption of arterial revascularization.

## Funding support and author disclosures

The authors have reported that they have no relationships relevant to the contents of this paper to disclose.
