# Supplementary material for: Bayesian Network Meta-Analysis of Long-Term Survival: Single vs Multiple vs Total Arterial Coronary Bypass Grafting
Source: JACC Adv. 2026 Aug 9;5(9):103178. doi: 10.1016/j.jacadv.2026.103178 (PMC13616513; doi:10.1016/j.jacadv.2026.103178)
Supplement: Supplementary file 1 [file mmc1.pdf]

Supplementary Table 1. Detailed search strategy (search has identified 3655 citations)

**For Ovid MEDLINE® and Epub Ahead of Print, In-Process, In-Data-Review & Other Non-Indexed Citations and Daily, EBM Reviews - Cochrane Central Register of Controlled Trials and Embase Classic +Embase <No Limit on Publication Year>**	
Searched on 09/03/2026	
1.	Coronary Artery Bypass/
2.	(coronary artery bypass* or CABG or coronary artery bypass graft* or coronary artery bypass surgery or coronary artery graft*).mp.
3.	(total* arter* or exclusiv* arter* or complete* arter* or total* arterial revasculari* or total* arterial graft* or TAR* or TAG*).mp.
4.	Radial Artery/
5.	(radial arter* or arteria radialis or radialis arter*).mp.
6.	Internal Mammary-Coronary Artery Anastomosis/
7.	(internal mammary arter* or internal thoracic arter* or bilateral internal mammary arter* or bilateral internal thoracic arter* or LIMA or RIMA or BIMA or LITA or RITA or BITA).mp.
8.	Gastroepiploic artery/
9.	(gastroepiploic arter* or GEA).mp.
10.	(multi-arterial or multiple arterial graft* or multiple arterial conduit* or multiple arterial revasculari* or MAG or composite arterial graft* or T-graft* or Y-graft*).mp.
11.	(surviv* or mortalit* or hazard ratio* or HR or MACCE or MACE or major adverse cardiac or KM or Kaplan Meier or Kaplan-Meier).mp.
12.	(hazard ratio* or HR* or KM or Kaplan Meier or Kaplan-Meier).mp.
13.	1 or 2
14.	3 or 4 or 5 or 6 or 7 or 8 or 9 or 10
15.	11 and 12 and 13 and 14
16.	limit 15 to english language
17.	Case Reports/
18.	comment/ or meeting abstract/ or "review"/
19.	16 not (17 or 18)
[mp=title, abstract, heading word, drug trade name, original title, device manufacturer, drug manufacturer, device trade name, keyword heading word, floating subheading word, candidate term word]	

Supplementary Table 2. Study-Level Hazard Ratios for Long-Term Mortality According to Arterial Grafting Strategy in Coronary Artery Bypass Grafting

Study	Intervention		Control		HR	95% CI	P-value	Data source	COF	Study Period
	Grafting Strategy	N	Grafting Strategy	N						
Aboul-Hassan (2024)	BIMA±RA or SVG	7283	Any SAG+SVG	7283	0.8	0.72–0.88	<0.001	Polish National Registry (KROK Registry) database	100.0%	2012-2020
Aboul-Hassan (2023)	BIMA+RA or 2 Arterial with sequential anastomoses	231	BIMA±RA +SVG	231	0.9	0.45–1.77	0.754	Poznan University	NA	2006-2020
Barili	BIMA±SVG	10449.1	SIMA±SVG	10420.7	0.87	0.78–1.00	0.05	PRIORITY Project	100.0%	2002-2008
Bisleri	BIMA±RA or LIMA+RA	175	LIMA+SVG	175	0.4	0.21–0.77	0.005	University of Brescia Medical School (Institutional Database)	95.9%	2005-2015
Browne	BIMA or LIMA+RA	509.5	LIMA+SVG	509.5	0.87	0.73–1.03	0.11	CORONARY Trial	NA	2006-2011
Hamilton	LIMA+RA	113	LIMA+SVG	112	0.74	0.52–1.04	0.08	RAPCO Trial	99.0%	1996-2005
Carrier	BIMA±SVG	1235	SIMA+SVG	5420	0.65	0.51–0.82	<0.0003	Régie de l'Assurance Maladie du Québec	99.0%	1995-2007
Chaubey	BIMA±SVG	407	SIMA+SVG	121	0.52	0.36–0.74	<0.001	Kings College Hospital	NA	1992-2014
Chikwe	Any MAG	3588	Any SAG	3588	0.8	0.72–0.90	0.01	New Jersey State Registry	99.0%	2005–2012
DiBacco	Any TAR	359	LIMA+SVG	359	0.55	0.38–0.76	<0.001	University of Brescia Medical School	95.1%	2005-2015
Dimitrova	LIMA+RA	283	LIMA+SVG	283	0.64	0.44–0.90	0.017	Beth Israel Medical Center (New York State Dept of Health)	NA	1995-2010
Garatti	Any TAR	209	LIMA+SVG	243	0.72	0.45–1.14	0.29	IRCCS Policlinico S. Donato	NA	1994-1996
Gaudino	≥2 arterial grafts	9512	1 arterial graft	9512	0.8	0.73–0.87	<0.001	New York's Cardiac Surgery Reporting System (CSRS)	NA	2005-2014
Gikandi	BIMA±SVG or IMA+RA±SVG	1261	IMA+SVG	24708	0.7	0.58–0.85	<0.001	Veterans Affairs (VA) Corporate Data Registry	NA	2005-2015
Goldstone	BIMA or LIMA+RA	5813	LIMA+SVG	5813	0.79	0.72–0.87	<0.001	California CABG Outcomes Reporting Program (CCORP)	NA	2006-2011

Guru	2 or 3 arterial grafts	5491	1 arterial graft	5491	0.8	0.72–0.99	<0.05	Cardiac Care Network Database	NA	1991-2001
Habib	IMA+RA±SVG	1416	IMA+SVG	1416	0.65	0.54–0.79	<0.001	Mercy Saint Vincent Medical Center Cardiac Surgery Database	NA	1996-2007
Hachiro	BIMA±SVG	599.72	LIMA+SVG	621.25	0.71	0.50–0.99	0.042	Shiga University of Medical Science	98.7%	2002-2022
Iribarne	BIMA±SVG	1297	SIMA±SVG	2197	0.79	0.69–0.91	0.001	Northern New England Cardiovascular Disease Study Group	100.0%	1992–2014
Jameie	≥2 arterial conduits	986	1 arterial conduit	22812	0.9	0.67–1.22	0.516	Tehran Heart Center	99.2%	2006–2016
Janiec	IMA+RA±SVG	1036	IMA+SVG	46343	1.01	0.89–1.14	0.92	SWEDEHEART Registry	NA	2001–2015
Joo	BIMA±RA ±SVG	366	LIMA+SVG	366	0.95	0.63–1.43	0.798	Yonsei Cardiovascular Hospital	98.4% (BITA), 97.8% (SITA)	2000–2009
Kieser	BIMA±SVG	1038	SIMA+SVG	4029	0.87	0.69–1.09	0.22	APPROACH / Alberta Cardiac Surgery Registry	NA	1995–2008
Kurlansky	BIMA±SVG	1226	SIMA+SVG	1226	0.81	0.73–0.91	0.00015	Florida Heart Research Institute	96.7%	1972–1994
Legare	Any TAR	1019	LIMA+SVG	3677	0.92	0.71–1.18	0.5	Maritime Heart Center (MHC) database	NA	1995–2003
Lin	LIMA+RA ±SVG	260	LIMA+SVG	260	0.76	0.60–0.97	0.03	Cedars-Sinai Medical Center	99.0%	1997–2001
Locker	≥2 arterial conduits	801	LIMA+SVG	801	0.78	0.66–0.92	0.003	Mayo Clinic	96.0%	1993-2009
Pu	≥2 arterial conduits	5580	LIMA+SVG	5351	0.79	0.72–0.87	<0.001	British Columbia Registry	NA	2000-2014
Qureshi	LIMA+ RA/BIMA±RA	1187	LIMA+SVG	1187	0.75	0.64–0.88	<0.001	Nottingham City Hospital	NA	2000–2018
Raja	Any TAR	580	Any Non-TAR	806	0.91	0.70–1.16	0.89	Harefield Hospital	100.0%	1998-2008
Ren	MAG with no SVG	11137	MAG with SVG	11137	0.85	0.80–0.91	<0.001	Australian and New Zealand Society of Cardiac and Thoracic Surgeons Database	100.0%	2001-2020
Rocha	≥2 arterial conduits	8629	1 arterial conduit	8629	0.8	0.73–0.88	<0.01	CorHealth Registry	NA	2008-2016
Rosenblum	≥2 arterial conduits	2624	1 arterial conduit	14068	0.77	0.67–0.89	0.0004	The Emory University	NA	2002-2016
Samadashvili	≥2 arterial conduits	10828	1 arterial conduit	10828	0.86	0.79–0.93	<0.001	New York's Cardiac Surgery Reporting System (CSRS) and New	100.0%	2005-2014

								York's Vital Statistics file		
Schwann	≥2 arterial conduits	3678	1 arterial conduit	6782	0.87	0.80–0.95	0.002	Multicentre	NA	1994-2011
Thuijs	≥2 arterial conduits	465	1 arterial conduit	1001	0.74	0.55–0.98	0.038	SYNTAX Extended Survival Study (SYNTAXES)	NA	2017-2019
Toumpoulis	BIMA±SVG	490	SIMA+SVG	490	0.89	0.69–1.14	0.343	St. Luke's–Roosevelt Hospital Center	NA	1992-2002
Tranbaugh	LIMA+RA ±SVG	4577	LIMA+SVG	7073	0.82	0.75–0.89	<0.001	Multicentre	NA	1995-2012
Yamaguchi	≥2 arterial conduits	577	1 arterial conduit	577	0.75	0.60–0.94	0.0132	Saitama Medical Center, Jichi Medical University	95.6%	1990-2014
Zacharias	BIMA/IMA +RA or bilateral RA	562	IMA+SVG	3638	0.6	0.48–0.95	<0.001	The Society of Thoracic Surgeons (STS) national database	NA	1992-2006
Zhang	≥2 arterial conduits	243	1 arterial conduit	676	0.77	0.47–1.24	0.29	Jiangsu Province CABG Registry	96.6%	2013-2019
Zhu	LIMA+RA ±SVG	197	IMA+SVG	181	0.42	0.20–0.88	0.02	Ruijin Hospital	90.9%	1999-2005
Benedett	LIMA+RA ±SVG	2007	LIMA+SVG	2007	0.86	0.74–0.99	0.04	National Institute for Cardiovascular Outcomes Research (NICOR) NACSA	NA	1996-2015
Gatti	BIMA±SVG	247	SIMA+SVG	247	0.71	0.55–0.91	0.005	University Hospital of Trieste	100.0%	1999-2016
Popovic	BIMA±SVG	130	LIMA+SVG	130	1.02	0.68–1.56	0.8	CHU Nancy	NA	1996-2009
Jeong	LIMA+RIMA	292	LIMA+SVG	292	0.80	0.45–1.40	/	Samsung Medical Center	NA	2001-2010
Medalion	BIMA	1045	LIMA+SVG	582	0.83	0.71–0.97	<0.005	Israeli National Registry	98.0%	1996-2008
Grau	BIMA	928	LIMA+SVG	928	0.67	0.54–0.84	<0.0001	The Valley Heart and Vascular Institute	NA	1994-2010
Kunihara	LIMA+RA	104	LIMA+SVG	104	0.84	0.56–1.25	/	The Cardiovascular Institute Tokyo	NA	1994-2001
Navia	BIMA	2098	LIMA+RA +SVG	1242	0.79	0.65–0.99	0.0356	Institute Cardiovascular of Buenos Aires	NA	1996-2014
Sabik	Any MAG	100404	Any SAG	100404	0.86	0.85–0.88	0.0001	Society of Thoracic Surgery Database	NA	2008-2019
Dayan	Any MAG	2761	Any SAG	2761	0.88	0.79–0.97	0.013	National Resources Fund (NRF) of Uruguay Database	NA	2002-2022

Al-Holy	Any MAG	662	Any SAG	662	0.97	0.78–1.20	0.954	Patient Analysis and Tracking System (PATS)	100.0%	1996-2023
Taggart	Any TAR	843	Any SAG	1084	0.68	0.48–0.96	0.03	Arterial Revascularisation (ART) Trial	94.4%	2004-2007

N, number of patients; COF, completeness of follow-up; ART, Arterial Revascularisation Trial; BIMA, bilateral internal mammary artery; BITA, bilateral internal thoracic artery; CABG, coronary artery bypass grafting; CCORP, California CABG Outcomes Reporting Program; CHU, Centre Hospitalier Universitaire; CI, confidence interval; CSRS, Cardiac Surgery Reporting System; HR, hazard ratio; IMA, internal mammary artery; IRCCS, Istituto di Ricovero e Cura a Carattere Scientifico; KROK, National Registry of Cardiac Surgery Procedures; LIMA, left internal mammary artery; MAG, multiple arterial grafting; MHC, Maritime Heart Center; NACSA, National Adult Cardiac Surgery Audit; NRF, National Resources Fund; OPCAB, off-pump coronary artery bypass; PATS, Patient Analysis and Tracking System; RA, radial artery; RAPCO, Radial Artery Patency and Clinical Outcomes; RIMA, right internal mammary artery; SAG, single arterial grafting; SIMA, single internal mammary artery; SITA, single internal thoracic artery; STS, Society of Thoracic Surgeons; SVG, saphenous vein graft; SYNTAXES, SYNTAX Extended Survival Study; TAR, total arterial revascularisation; VA, Veterans Affairs.

Supplementary Table 3. Baseline Characteristics of Patients in the Intervention Groups Across Included Studies

Study	Number of grafts	Age (year)	Males	Smoking (%)	Hypertension (%)	Dyslipidemia (%)	Diabetes (%)	PVD (%)	MI (%)	COPD (%)	OPCAB (%)
Aboul-Hassan (2024)	2.6±0.72	59.7±8.70	7283 (100)	1787 (24.5)	6127 (84.1)	4822 (66.2)	2107 (28.9)	1130 (15.5)	2163 (29.7)	NA	4354 (59.8)
Aboul-Hassan (2023)	3.1±0.2	57.1±8.63	18 (12.1)	66(28.6)	205(88.7)	105(45)	65(28.1)	25(10.8)	123(53.2)	NA	61 (26.4)
Barili	NA	67.1±9.5	2040.1 (19.6)	NA	NA	NA	3418.1 (32.8)	2100.2 (20.2)	2614 (25.1)	979.9 (9.4)	2894.7 (27.7)
Bisleri	2.5±0.5	76±4	128 (73.2)	NA	137 (78.2)	106 (60.5)	82 (46.8)	30 (17.1)	79 (45.1)	24 (13.7)	62 (36.9)
Browne	NA	67.2±5.6	428 (83.9)	242 (47.3)	371 (72.9)	NA	240 (47.1)	51 (9.9)	178 (34.9)	NA	260 (51.1)
Hamilton	3.2±0.9	72.6	91 (81)	75 (66)	68 (60)	NA	50 (44)	NA	43 (38)	NA	NA
Carrier	NA	61±9.0	1036 (84.0)	NA	567 (46.0)	671 (54.0)	254 (21.0)	NA	218 (18.0)	NA	NA
Chaubey	NA	53.2±6.4	329 (81.0)	105 (26.0)	213 (52.0)	NA	61 (15%)	NA	170 (42.0)	38 (9.0)	NA
Chikwe	3.6±1.0	60.9±10.4	3043 (84.8)	2214 (61.7)	3204 (89.3)	NA	1457 (40.6)	499 (13.9)	1572 (43.8)	779 (21.7)	1769 (49.3)
DiBacco	2.6±0.4	70.4±9.6	283 (78.8)	NA	223 (62.1)	183 (50.9)	136 (38.3)	50 (13.9)	153 (42.6)	51 (14.5)	126 (35.1)
Dimitrova	3.5±0.9	61±8.6	0	NA	214 (76.0)	NA	151 (53)	32 (11.3)	116 (41.0)	77 (27.2)	3 (0.9)
Garatti	3.2±0.8	48±8	199 (95.0)	155 (74.0)	75 (36.0)	140 (67.0)	31 (15.0)	48 (23.0)	144 (69.0)	8 (4.0)	0 (0.0)
Gaudino	NA	61.2 ± 9.4	NA	NA	NA	NA	2915 (30.6)	887 (9.3)	1449 (15.2)	1336 (14.0)	1895 (19.9)
Gikandi	NA	61.1 ± 7.1	1256 (99.6)	NA	1176 (93.3)	NA	568 (45.0)	253 (20.1)	NA	317 (25.1)	0 (0.0)
Goldstone	NA	62.0 ± 10.4	4960 (85.3)	NA	4702 (80.9)	NA	2051 (35.3)	554 (9.5)	1250 (21.5)	856 (14.8)	NA
Guru	NA	NA	4887 (89.0)	NA	NA	NA	1153 (21.0)	439 (8.0)	NA	384 (7.0)	NA
Habib	2±0	NA	NA	NA	NA	NA	NA	NA	NA	NA	NA
Hachiro	2.7±0.5	69.1 ± 9.7	482.9 (80.5)	363.8 (60.7)	430.3 (71.8)	324.5 (54.1)	324.5 (54.1)	37.6 (6.3)	194.5 (32.4)	NA	600 (100.0)
Iribarne	NA	NA	1060 (81.7)	NA	NA	NA	213 (16.4)	208 (16.0)	218 (16.8)	105 (8.1)	124 (9.6)
Jameie	3.9 ± 0.9	56.7 ± 9.2	905 (92.0)	236 (24.0)	392 (39.8)	668 (67.9)	287 (29.2)	15 (1.5)	150 (15.3)	24 (2.4)	68 (7.0)
Janiec	NA	64.5 ± 9.7	759 (73.3)	NA	NA	NA	212 (20.7)	70 (10.2)	267 (39.1)	39 (5.7)	74 (7.1)
Joo	3.3±0.7	60.7 ± 8.1	279 (76.2)	198 (54.1)	192 (52.5)	NA	138 (37.8)	63 (17.2)	119 (32.5)	19 (5.2)	NA
Kieser	NA	58.0 ± 9.1	NA	433 (41.7)	678 (65.3)	828 (79.8)	289 (27.8)	77 (7.4)	435 (41.9)	122 (11.8)	NA

Kurlansky	NA	NA	NA	NA	NA	NA	NA	NA	NA	NA	NA
Legare	3.1 ± 0.9	60.2 ± 9.7	818 (80.0)	732 (72.0)	629 (62.0)	902 (88.0)	347 (34.0)	138 (14.0)	36 (4.0)	120 (12.0)	0 (0.0)
Lin	NA	70.6 ± 8.7	181 (69.6)	NA	169 (65.0)	NA	101 (38.8)	57 (21.9)	NA	33 (12.7)	43 (16.5)
Locker	NA	59.3 ± 8.9	684 (85.4)	NA	546 (68.2)	NA	159 (19.9)	101 (12.6)	218 (27.2)	58 (7.2)	NA
Pu	NA	60.0 ± 8.7	4994 (89.5)	988 (17.7)	4224 (75.7)	NA	1650 (29.6)	559 (10.0)	3085 (55.3)	816 (14.6)	NA
Qureshi	NA	68	947 (80.0)	789 (66.5)	754 (63.5)	NA	256 (22.0)	137 (11.5)	NA	57 (4.8)	336 (28.3)
Raja	3.1±0.9	NA	477 (82.2)	NA	364 (62.7)	510 (87.8)	205 (35.3)	72 (12.4)	5 (0.9)	62 (10.7)	580 (100)
Ren	3.3±0.8	66.2±10.0	18287 (80.3)	14694 (64.5)	18313 (80.4)	18458 (81.1)	8522 (37.4)	2443 (10.7)	11931 (52.4)	2705 (11.9)	1298 (5.7)
Rocha	3.8±0.8	63.9±10.0	7188 (83.3)	4955 (57.4)	6209 (72.0)	6367 (73.8)	3150 (36.5)	873 (10.1)	4859 (56.3)	526 (6.1)	1833 (21.2)
Rosenblum	3.2	58.96±0.21	NA	1198 (55.8)	1764 (82.1)	1843 (85.8)	753 (35.1)	227 (10.6)	579 (27.0)	61 (2.8)	NA
Samadashvili	NA	NA	8904 (82.2)	NA	NA	NA	3406 (31.5)	1102 (10.2)	4465 (41.2)	1676 (15.5)	1915 (17.7)
Schwann	3.7±0.8	60.9±9.7	2247 (76.9)	NA	2240 (76.7)	NA	1090 (37.3)	297 (10.2)	NA	545 (18.7)	NA
Thuijs	2.8±0.7	62.3±9.7	401 (86.2)	319 (69.0)	344 (74.9)	361 (78.6)	141 (30.3)	49 (10.5)	128 (28.0)	34 (7.3)	101 (21.7)
Toumpoulis	3.7±0.9	63.6±9.9	270 (55.1)	72 (14.7)	405 (82.7)	NA	490 (100)	128 (26.1)	266 (54.3)	76 (15.5)	27 (5.5)
Tranbaugh	3.7±0.9	60.3±9.7	3544 (77.4)	NA	3362 (73.5)	NA	1702 (37.2)	414 (9.0)	2325 (50.8)	781 (17.1)	138 (3.0)
Yamaguchi	3.27±0.90	64.8±9.7	479 (83.0)	NA	267 (46.3)	214 (37.1)	0 (0)	39 (6.8)	187 (32.4)	NA	NA
Zacharias	NA	60±10.5	437 (71.4)	NA	NA	NA	192 (31.4)	91 (14.9)	301 (49.2)	100 (16.4)	51 (8.3%)
Zhang	NA	63.2±9.1	198 (81.5)	76 (31.3)	140 (57.6)	NA	80 (32.9)	19 (7.8)	75 (30.9)	9 (3.7)	88 (36.2)
Zhu	3.3±0.88	55.9±7.5	183 (88.0)	118 (56.7)	157 (75.5)	114 (54.8)	71 (34.1)	3 (1.4)	101 (48.6)	14 (6.7)	208 (100)
Benedett	2.80±0.70	NA	1740 (87)	315 (16)	1368 (68)	NA	358 (18)	148 (7)	900 (45)	181 (9)	NA
Gatti	NA	NA	0 (0)	NA	195 (78.9)	NA	87 (35.2)	NA	56 (22.7)	13 (5.3)	NA
Popovic	2.5±0.6	60.6±9.9	114 (87.7)	NA	70 (53.8)	79 (60.8)	53 (40.8)	44 (33.8)	18 (13.8)	24 (18.5)	3/167 (1.8)
Jeong	4.1±0.8	63±9	201 (69)	65 (22)	186 (64)	87 (30)	141 (48)	16 (6)	48 (16)	16 (6)	292 (100)
Medalion	NA	NA	763 (73.0)	NA	NA	NA	337 (32.2)	224 (21.4)	453 (43.3)	57 (5.5)	316 (30.3)
Grau	3.6	60.9±9	828 (89.2)	598 (64.4)	631 (68.0)	625 (67.3)	100 (10.8)	84 (4.5)	416 (44.8)	17 (1.8)	454 (48.9)
Kunihara	NA	64.0 ± 8.0	75 (72.0)	60 (58.0)	93 (89.0)	78 (75.0)	104 (100.0)	21 (20.0)	36 (35.0)	NA	0 (0.0)
Navia	NA	65.4±8.9	419 (86.4)	291 (60)	369 (76)	355 (73)	131 (27)	22 (4.5)	126 (26)	23 (4.7)	477 (98.4)
Sabik	3.6 ± 1.0	60.3 ± 10.1	84841 (84.5)	NA	84339 (84.0)	NA	37752 (37.6)	11044 (11.0)	28709 (28.6)	16567 (16.5)	NA
Dayan	NA	57.1 ± 8.8	NA	1055 (38.2)	1727 (62.6)	1588 (57.5)	727 (26.3)	141 (5.1)	666 (24.1)	NA	752 (27.2)
Al-Holy	NA	60.8 ± 9.2	NA	NA	458 (69.2)	523 (79.0)	NA	70 (10.6)	NA	64 (9.7)	NA
Taggart	NA	64 ± 9	NA	NA	662 (78.5)	785 (93.2)	201 (23.8)	56 (6.7)	366 (43.4)	11 (1.3)	330 (39.1)

This table summarizes the baseline demographic, clinical, and operative characteristics of patients assigned to the intervention groups across the included studies. Reported variables include mean number of grafts, age, sex distribution, and prevalence of major cardiovascular and operative risk factors, including smoking, hypertension, dyslipidemia, diabetes, peripheral vascular disease, prior myocardial infarction, chronic obstructive pulmonary disease, and off-pump coronary artery bypass grafting. Values are presented as mean ± standard deviation or number (percentage), as reported in the original studies. Data are shown for the intervention cohorts only and are intended to describe the clinical profile of patients receiving the study treatment strategy. PVD, peripheral vascular disease; MI, myocardial infarction; COPD, chronic obstructive pulmonary disease; OPCAB, off-pump coronary artery bypass.

Supplementary Table 4. Baseline Characteristics of Patients in the Control Groups Across Included Studies

Study	Number of grafts	Age (year)	Males	Smoking (%)	Hypertension (%)	Dyslipidemia (%)	Diabetes (%)	PVD (%)	MI (%)	COPD (%)	OPCAB (%)
Aboul-Hassan (2024)	2.6±0.79	59.7±8.33	7283 (100)	1818 (25.0)	6125 (84.1)	4812 (66.1)	2149 (29.5)	1172 (16.1)	2092 (28.7)	NA	4406 (60.5)
Aboul-Hassan (2023)	3.1±0.2	58.4±8.42	47(12.5)	96(22.6)	357(84.2)	185(43.6)	104(24.5)	31(7.3)	123(53.2)	NA	NA
Barili	NA	67.2±9.5	2055.7 (19.7)	NA	NA	NA	3337.2 (31.9)	2177 (20.8)	2579.5 (24.7)	958 (9.4)	2894.7 (27.7)
Bisleri	2.6±0.41	79±7	126 (72.0)	NA	126 (72.0)	102 (58.2)	87 (49.7)	30 (17.1)	66 (37.7)	24 (13.7)	36 (20.6)
Browne	NA	67.2±2.7	428 (83.9)	242 (47.3)	371 (72.9)	NA	240 (47.1)	51 (9.9)	178 (34.9)	NA	260 (51.1)
Hamilton	3.3±0.7	73.1	91 (81)	76 (68)	78 (70)	NA	52 (46)	NA	36 (32)	NA	NA
Carrier	NA	68±8.0	3841 (71.0)	NA	2821 (48.0)	2468 (46.0)	1696 (31.0)	NA	1032 (19.0)	NA	NA
Chaubey	NA	53.9±5.3	89 (74.0)	30 (25.0)	47 (39.0)	NA	35 (29.0)	NA	50 (41.0)	13 (11.0)	NA
Chikwe	3.6±1.0	61.1±10.2	3050 (85.0)	2228 (62.1)	3233 (90.1)	NA	1446 (40.3)	499 (13.9)	1579 (44.0)	786 (21.9)	1816 (50.6)
DiBacco	2.6±0.6	69.7±9.4	272 (75.8)	NA	215 (59.9)	178 (49.6)	133 (36.0)	50 (13.9)	137 (38.1)	43 (12.2)	76 (21.1)
Dimitrova	3.5±0.9	61±8.8	0	NA	214 (76.0)	NA	155 (55.0)	31 (10.9)	119 (42.0)	77 (27.2)	7 (2.6)
Garatti	3.9 ± 0.7	50 ± 7	233 (96.0)	182 (75.0)	104 (43.0)	158 (65.0)	34 (14.0)	27 (11.0)	168 (69.0)	7 (3.0)	0 (0.0)
Gaudino	NA	61.1 ± 9.5	NA	NA	NA	NA	2999 (31.5)	933 (9.8)	1452 (15.3)	1443 (15.2)	1915 (20.1)
Gikandi	NA	62.9 ± 6.5	24499 (99.2)	NA	23251 (94.1)	NA	13468 (54.5)	5375 (21.8)	NA	8364 (33.9)	0 (0.0)
Goldstone	NA	62.5 ± 10.4	4897 (84.2)	NA	4748 (81.7)	NA	2066 (35.5)	573 (9.9)	1250 (21.5)	856 (14.8)	NA
Guru	NA	NA	4887 (89.0)	NA	NA	NA	1153 (21.0)	439 (8.0)	NA	384 (7.0)	NA
Habib	2±0	NA	1416 (100.0)	NA	NA	NA	NA	NA	NA	NA	NA
Hachiro	2.2±0.4	68.9 ± 10.4	505.9 (81.4)	378.8 (60.9)	435.1 (70.0)	339.0 (54.5)	357.1 (57.4)	37.2 (6.0)	179.0 (28.8)	NA	621 (100.0)
Iribarne	NA	NA	1051 (81.0)	NA	NA	NA	217 (16.7)	219 (16.9)	214 (16.5)	112 (8.6)	124 (9.6)
Jameie	3.5 ± 0.8	65.5 ± 9.8	16676 (73.1)	3968 (17.4)	12957 (56.8)	14785 (64.8)	9270 (40.6)	486 (2.1)	4184 (18.4)	855 (3.8)	1521 (6.7)
Janiec	NA	66.4 ± 8.4	37464 (80.8)	NA	NA	NA	11077 (24.3)	3234 (6.8)	15301 (40.6)	2551 (6.9)	1103 (2.4)
Joo	3.2±0.8	60.1 ± 8.6	288 (78.7)	207 (56.6)	195 (53.3)	NA	144 (39.3)	63 (17.2)	114 (31.1)	16 (4.4)	NA
Kieser	NA	67.6 ± 9.5	NA	1847 (45.8)	2629 (65.3)	2652 (65.8)	1057 (26.2)	441 (11.0)	2097 (52.1)	578 (14.4)	NA
Kurlansky	NA	NA	NA	NA	NA	NA	NA	NA	NA	NA	NA
Legare	3.2 ± 0.8	65.8 ± 9.8	2689 (73.0)	2565 (70.0)	2368 (64.0)	2703 (74.0)	1360 (37.0)	623 (17.0)	208 (6.0)	429 (14.0)	0 (0.0)
Lin	NA	70.9 ± 9.8	183 (70.4)	NA	175 (67.3)	NA	91 (33.5)	52 (20.0)	NA	39 (15.0)	47 (18.1)

Locker	NA	59.3 ± 10.2	687 (85.8)	NA	551 (68.8)	NA	171 (21.3)	103 (12.9)	233 (29.1)	59 (7.4)	NA
Pu	NA	60.3 ± 8.5	12961 (89.4)	2522 (17.4)	11017 (76.0)	NA	4276 (29.5)	1450 (10.0)	8002 (55.2)	2131 (14.7)	NA
Qureshi	NA	67	959 (80.4)	794 (67.0)	734 (62.0)	NA	260 (22.0)	148 (12.5)	NA	65 (5.5)	329 (27.7)
Raja	3.4±0.8	NA	525 (65.1)	NA	520 (64.5)	641 (79.5)	319 (39.6)	147 (18.3)	9 (1.1)	147 (18.3)	806 (100)
Ren	3.2±0.9	66.6±10.1	18218 (80.0)	14824 (65.1)	18513 (81.3)	18449 (81.0)	8723 (38.3)	2484 (10.9)	12349 (54.2)	2824 (12.4)	1130 (5.0)
Rocha	3.5±0.7	63.8±9.9	7202 (83.5)	5021 (58.2)	6160 (71.4)	6343 (73.5)	3110 (36.0)	896 (10.4)	4798 (55.6)	537 (6.2)	1776 (20.6)
Rosenblum	2.6	64.65±0.11	NA	4138 (45.9)	7900 (87.6)	7603 (84.3)	3846 (42.7)	1320 (14.6)	1709 (19.0)	748 (8.3)	NA
Samadashvili	NA	NA	8974 (82.9)	NA	NA	NA	3430 (31.7)	1076 (9.9)	4467 (41.3)	1615 (14.9)	1915 (7.7)
Schwann	3.6±0.8	67.9±9.7	3471 (66.3)	NA	4109 (78.4)	NA	2024 (38.6)	847 (16.2)	NA	1378 (26.3)	NA
Thuijs	2.8±0.8	66.5±9.2	777 (77.6)	657 (66.1)	742 (74.9)	751 (76.2)	361 (36.1)	128 (12.8)	351 (35.7)	89 (8.9)	146 (14.5)
Toumpoulis	3.3±0.9	64.5±9.4	275 (56.1)	81 (16.5)	386 (78.8)	NA	490 (100)	150 (30.6)	292 (59.6)	85 (17.3)	35 (7.1)
Tranbaugh	3.4±0.9	67.4±9.9	4625 (65.4)	NA	5400 (76.3)	NA	2704 (38.2)	1129 (16.0)	4056 (57.3)	1804 (25.5)	249 (3.5)
Yamaguchi	3.27±0.93	65.8±9.8	471 (81.6)	NA	295 (51.1)	221 (38.3)	0 (0)	42 (7.3)	195 (33.8)	NA	NA
Zacharias	NA	65.3±10.3	2779 (67.3)	NA	NA	NA	1405 (34.0)	567 (13.7%)	2416 (58.5)	791 (19.1)	95 (2.3)
Zhang	NA	64.5±8.9	552 (81.7)	222 (32.8)	399 (59.0)	NA	229 (33.9)	39 (5.8)	288 (33.7)	24 (3.6)	270 (39.9)
Zhu	3.3±0.7	56.2±6.9	183 (88.0)	121 (58.1)	170 (81.7)	125 (60.1)	78 (37.5)	12 (5.7)	85 (40.9)	16 (7.7)	208 (100)
Benedett	2.81±0.70	NA	1717 (86)	345 (17)	1365 (68)	NA	394 (20)	161 (8)	926 (46)	184 (9)	NA
Gatti	NA	NA	0 (0)	NA	193 (78.1)	NA	90 (36.4)	NA	54 (21.9)	13 (5.3)	NA
Popovic	2.5±0.7	60.5±10.0	111 (85.4)	NA	73 (56.2)	82 (63.1)	54 (41.5)	45 (34.6)	21 (16.2)	23 (17.7)	18 (6.8)
Jeong	4.6±1.0	64±9	201 (69)	70 (24)	187 (64)	96 (33)	143 (49)	21 (7)	42 (14)	10 (3)	292 (100)
Medalion	NA	NA	386 (66.3)	NA	NA	NA	223 (38.3)	105 (18)	242 (41.6)	75 912.9)	167 (28.7)
Grau	3.4	62.1±9	829 (89.3)	635 (68.4)	641 (69.1)	605 (65.2)	101 (10.9)	92 (5.0)	420 (45.3)	20 (2.1)	476 (51.3)
Kunihara	NA	65.0 ± 7.0	72 (69.0)	56 (54.0)	88 (85.0)	79 (76.0)	104 (100.0)	26 (25.0)	36 (35.0)	NA	0 (0.0)
Navia	NA	65.5±9.5	418 (86.2)	280 (57)	370 (76)	347 (71)	141 (29)	11 (2.3)	125 (25.8)	22 (4.5)	477 (98.4)
Sabik	3.5 ± 0.9	60.2 ± 10.3	84440 (84.1)	NA	84339 (84.0)	NA	37240 (37.1)	11245 (11.2)	29114 (29.0)	16767 (16.7)	NA
Dayan	NA	57.0 ± 9.3	NA	1047 (37.9)	1737 (62.9)	1614 (58.5)	729 (26.4)	138 (5.0)	622 (22.5)	NA	706 (25.6)
Al-Holy	NA	61.5 ± 9.0	NA	NA	464 (70.1)	518 (78.2)	NA	68 (10.3)	NA	61 (9.2)	NA
Taggart	NA	64 ± 9	NA	NA	852 (78.6)	1016 (93.7)	253 (23.3)	73 (6.7)	481 (44.4)	31 (2.9)	413 (38.1)

This table summarizes the baseline demographic, clinical, and operative characteristics of patients in the control groups across the included studies. Reported variables include the mean number of grafts, age, sex distribution, and the prevalence of major cardiovascular risk factors and comorbidities, including smoking, hypertension, dyslipidaemia, diabetes, peripheral vascular disease, prior myocardial infarction, chronic obstructive pulmonary disease, and use of off-pump coronary artery bypass grafting.

Values are presented as mean ± standard deviation or number (percentage), as reported in the original studies. These data describe the clinical profile of patients receiving the comparator revascularisation strategy in each study. PVD, peripheral vascular disease; MI, myocardial infarction; COPD, chronic obstructive pulmonary disease; OPCAB, off-pump coronary artery bypass.

Supplementary Table 5. GRADE Certainty-of-Evidence Quality Assessment for Network Estimates of Long-Term All-Cause Mortality

Comparison	Effect estimate	Starting certainty	Risk of bias	Inconsistency	Indirectness	Imprecision	Publication bias	Other considerations	Final certainty
**MAG vs SAG**	HR **0.83** (95% CrI 0.79–0.85)	Low†	Not serious	Not serious	Not serious	Not serious	Undetected	Large, consistent effect across 35 studies	**Moderate**
**TAR vs SAG**	HR **0.75** (95% CrI 0.70–0.79)	Low†	Not serious	Not serious	Not serious	Not serious	Undetected	Large effect; consistent direction across studies	**Moderate**
**TAR vs MAG**	HR **0.90** (95% CrI 0.85–0.96)	Low†	Not serious	Not serious	**Serious**	**Serious**	Undetected	Comparison relies heavily on indirect evidence; only 3 direct studies	**Low**

† Because most contributing evidence is from observational studies, GRADE starts at low certainty.

CrI, credible interval; CI, confidence interval; GRADE, Grading of Recommendations Assessment, Development and Evaluation; HR, hazard ratio; MAG, multiple arterial grafting; RCT, randomized controlled trial; SAG, single arterial grafting; TAR, total arterial revascularisation.

Supplementary Figure 1. Markov Chain Monte Carlo Trace and Posterior Density Plot

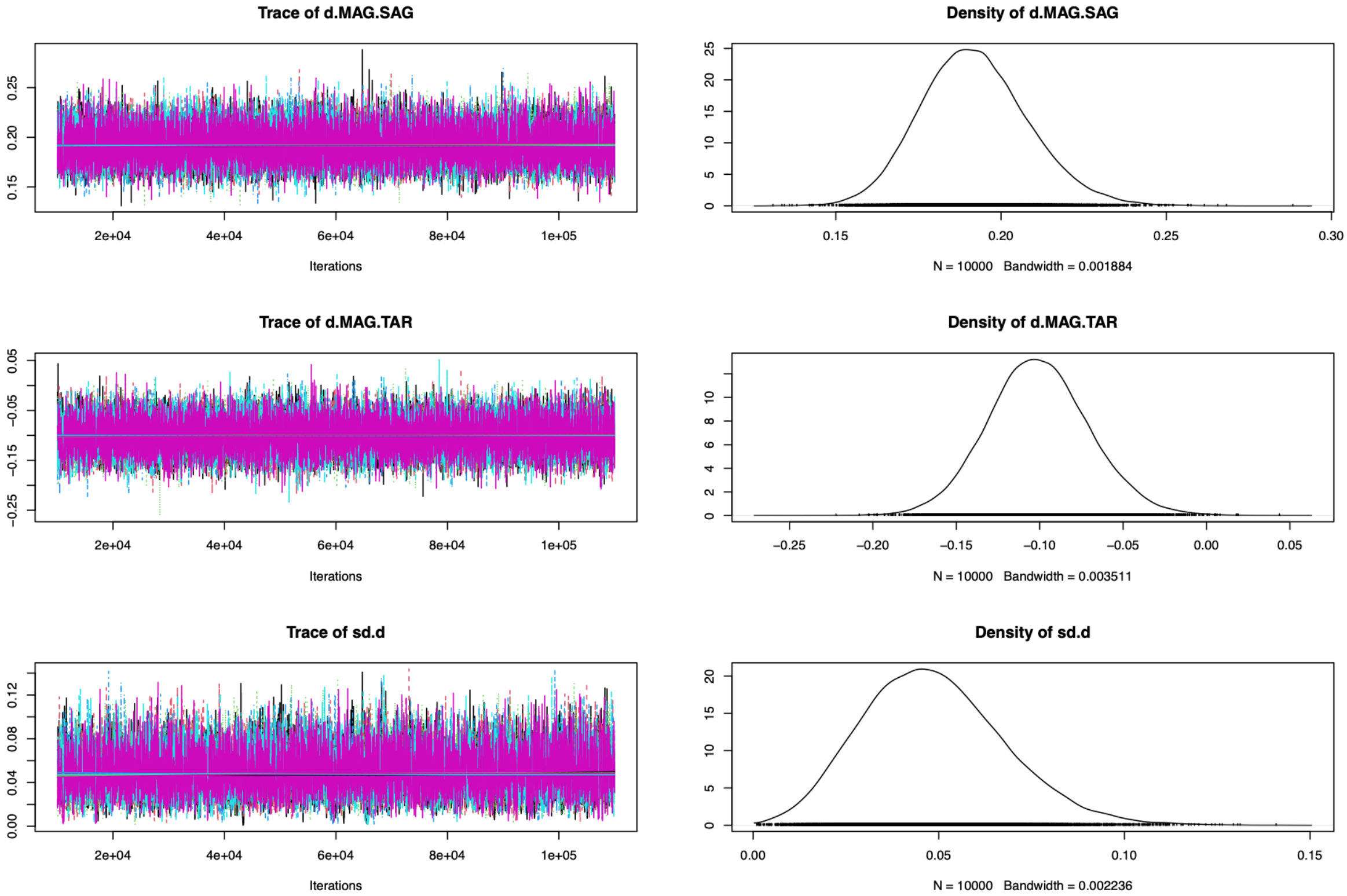

Trace plots and posterior density distributions from the Bayesian network meta-analysis showing convergence diagnostics for the estimated treatment effects and between-study heterogeneity. The left panels display the Markov chain trace plots across iterations for the relative treatment effects MAG versus SAG (d.MAG.SAG), MAG versus TAR (d.MAG.TAR), and the estimated between-study heterogeneity parameter (sd.d). The right panels show the corresponding posterior density distributions for these parameters. Stable trace plots without systematic trends and unimodal posterior density distributions indicate adequate mixing and convergence of the Markov chains. Results are based on 10,000 posterior samples after model adaptation and burn-in.

Supplementary Figure 2. Node-Splitting Analysis Assessing Consistency Between Direct and Indirect Evidence

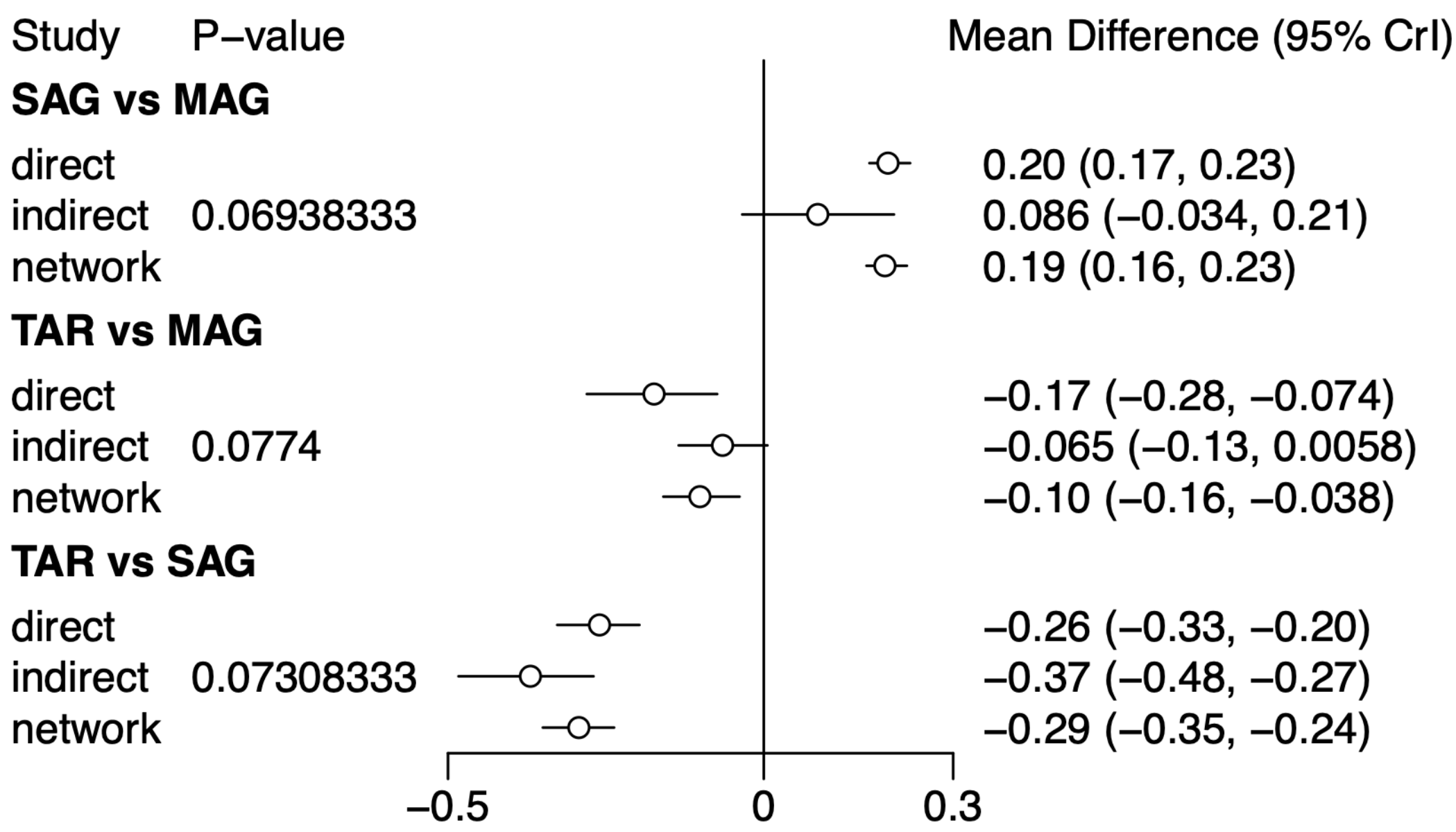

Forest plot from the node-splitting analysis evaluating the consistency between direct and indirect evidence in the Bayesian network meta-analysis. Mean difference is equivalent to log(HR). For each treatment comparison–SAG versus MAG, TAR versus MAG, and TAR versus SAG–the figure presents the effect estimates derived from direct evidence, indirect evidence, and the combined network estimate, with corresponding 95% credible intervals (CrIs). The P-values represent statistical tests for inconsistency between direct and indirect estimates. The vertical line represents the line of no effect. The similarity between direct and network estimates and non-significant inconsistency tests suggest agreement between sources of evidence in the network

Supplementary Figure 3. ROBINS-I + RoB-2 Risk of Bias Assessment

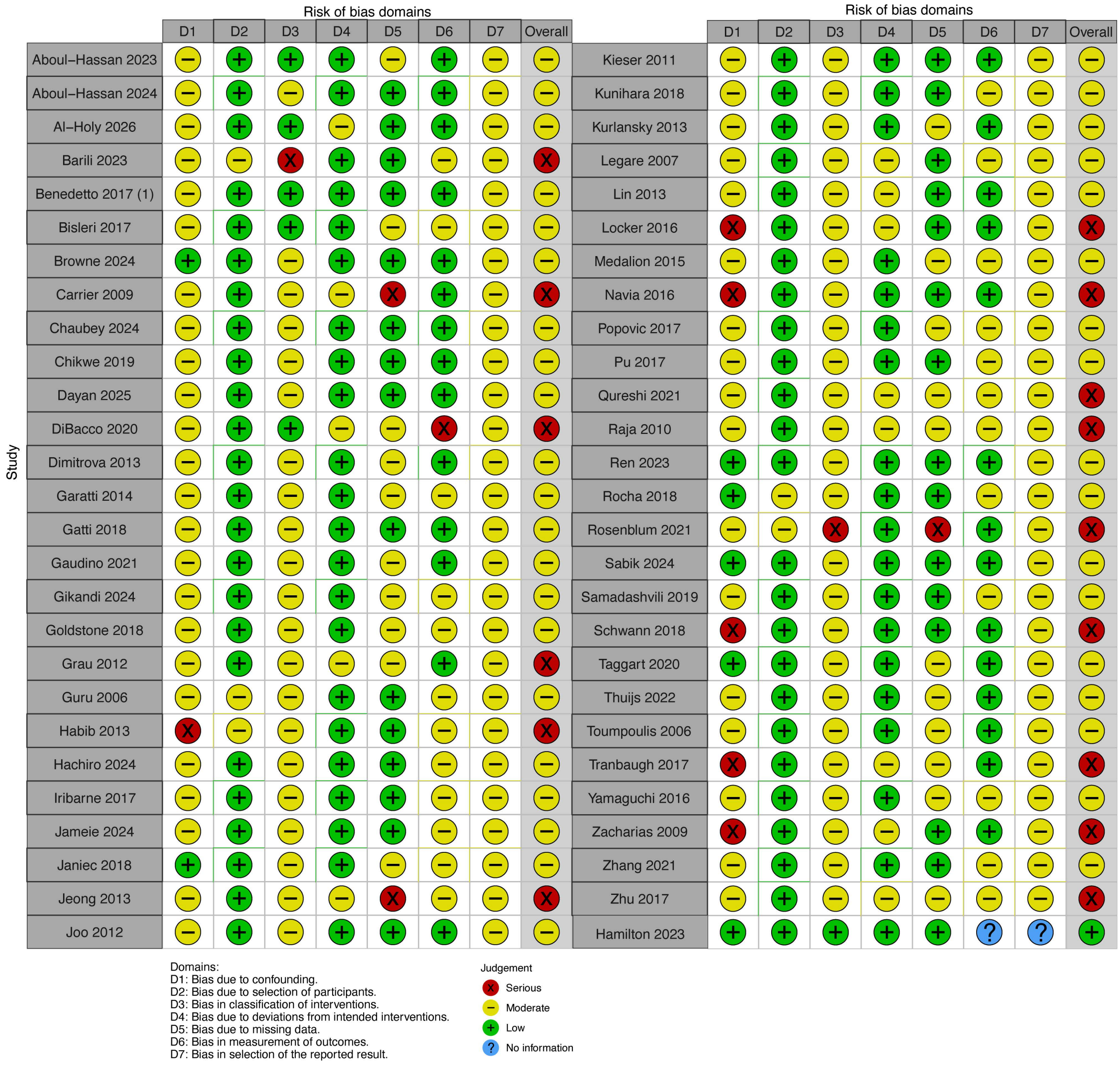

Domain-level risk-of-bias assessment of the included studies. Non-randomized studies were evaluated using the Risk Of Bias In Non-randomized Studies of Interventions (ROBINS-I) tool across the domains of confounding, selection of participants, classification of interventions, deviations from intended interventions, missing data, measurement of outcomes, and selection of the reported result, with overall judgements assigned according to ROBINS-I guidance. Because Hamilton 2023 was a randomized controlled trial, it was assessed separately using the revised Cochrane risk-of-bias tool for randomized trials (RoB 2), which evaluates 5 domains: bias arising from the randomization process, bias due to deviations from intended interventions, bias due to missing outcome data, bias in measurement of the outcome, and bias in selection of the reported result. ROBINS-I, Risk Of Bias In Non-randomized Studies of Interventions; RoB 2, revised Cochrane risk-of-bias tool for randomized trials; RCT, randomized controlled trial.
